# Ultrafine garlic powder alleviates non-alcoholic steatohepatitis by inhibiting hepatocyte ferroptosis and modulating ERK-dependent oxidative stress

**DOI:** 10.3389/fphar.2025.1711917

**Published:** 2025-11-19

**Authors:** Junhui Yu, Ningning Shao, Jianming Yang, Long Yang, Kaiwen Hong, Yuzhu Chen, Xiaoming Zhao, Hongzhi Yu, Tao Zhang, Jinrui Dong

**Affiliations:** 1 Academy of Medical Engineering and Translational Medicine, School of Medicine, Tianjin University, Tianjin, China; 2 Department of Basic Medicine, Haihe Hospital, Tianjin University, Tianjin, China; 3 Department of Blood Purifying, Taida Hospital, Tianjin University, Tianjin, China; 4 School of Pharmaceutical Science and Technology, Tianjin University, Tianjin, China; 5 Department of Computer Science, Lafayette College, Easton, PA, United States; 6 School of Materials Science and Engineering, Beihang University, Beijing, China

**Keywords:** ultrafine powder, NASH, hepatocyte, ferroptosis, fibrosis, ROS

## Abstract

**Introduction:**

Nonalcoholic steatohepatitis (NASH) is a chronic liver disease associated with oxidative stress and ferroptosis, leading to liver injury and fibrosis. Garlic, renowned for its antioxidant and hepatoprotective properties, is commonly used in traditional medicine. Ultrafine powder technology enhances the physicochemical properties of natural products, improving their bioavailability and efficacy. This study explores the protective effects of ultrafine garlic powder (UGP) on NASH and its underlying mechanisms.

**Methods:**

In this study, UGP was prepared by high-speed particle collision technology and compared with traditional garlic powder (TGP).

**Results:**

UGP exhibited a particle size (d(0.5) of approximately 4 μm) that is 30 times smaller than TGP, showing significantly smaller particle size and lower contact angle (UGP=59.775°vs TGP=83.545°). UGP also demonstrated improved solubility and dispersibility, along with an enrichment of key organosulfur compounds such as allicinAllicin, Alliin, SAC, and GSAC. In vitro, UGP significantly reduced palmitic acid-induced ROS production (n=4, p< 0.0001), prevented mitochondrial dysfunction (n=4, p=0.0035), and thereby reduced hepatic stellate cell activation. UGP lowered intracellular Fe2+ levels in hepatocytes from 68.72% to 34.48% (n=4) and significantly protected hepatocytes from erastin-induced ferroptosis (cell viability: UGP treatment 23.46% vs erastin stimulation 53.91%, n = 4, p <0.0001).In the acute liver fibrosis model induced by methionine- and choline-deficient diet supplemented with 60 kcal% fat (CDHF), UGP significantly improved liver histology. Compared to the model group, the liver/body weight ratio of mice was significantly restored (n=4, p<0.0001), and histopathological staining showed a substantial improvement in liver damage. Biochemically, UGP significantly reduced the levels of liver collagen, triglycerides, and cholesterol (n = 6, p < 0.0001). Fibrosis-related mRNA and pro-inflammatory mRNA expression were significantly decreased. Western blot results showed that UGP significantly inhibited the activation of p-ERK signaling. In the chronic liver fibrosis model induced by a diet with 60 kcal% fat (HFD), UGP demonstrated similar therapeutic effects. RNA-seq analysis revealed that UGP modulated key pathways, including fatty acid metabolism and the MAPK signaling pathway, and suppressed ROS production, further highlighting its therapeutic potential in NASH treatment.

**Discussion:**

Taken together, these data suggest that UGP alleviates NASH by inhibiting hepatocyte ferroptosis and modulating ERK-dependent oxidative stress, supporting its potential as a therapeutic agent.

## Graphical Abstract

**Figure:**
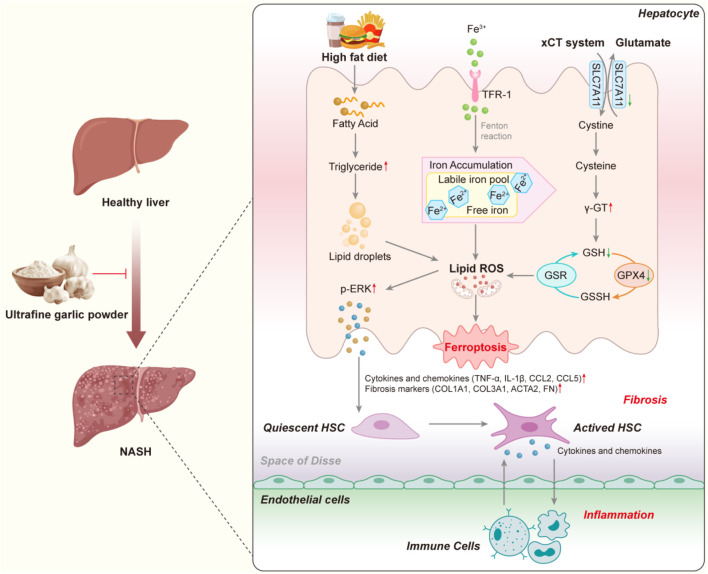
Schematic representation of the mechanism by which UGP exerts its beneficial effects on NASH by inhibiting the onset of ferroptosis. (ACTA2: Actin alpha cardiac muscle 2; CCL2: C-C motif chemokine ligand 2; CCL5: C-C motif chemokine ligand 5; COL1A1: Collagen type I alpha 1 chain; COL3A1: Collagen type III alpha 1 chain; FN: Fibronectin; GSH: Glutathione; GPX4: Glutathione peroxidase 4; GSSH: Glutathione disulfide; GSR: Glutathione reductase; HSC: Hepatic stellate cell; IL-1β: Interleukin 1 beta; NASH: Non-alcoholic steatohepatitis; ROS: Reactive oxygen species; SLC7A11: Solute carrier family 7 member 11; TNF-α: Tumor necrosis factor alpha; TFR-1: Transferrin receptor 1).

## 1 Introduction

Nonalcoholic fatty liver disease (NAFLD) is a chronic liver disorder with rising global prevalence, driven by environmental, nutritional, metabolic, and genetic factors (Rich et al., 2018; Marjot et al., 2020; Kechagias et al., 2008; Loomba et al., 2015), affecting an estimated 32.4% of adults by 2022 (Riazi et al., 2022). Excessive lipid accumulation in hepatocytes promotes inflammation and liver injury, contributing to NAFLD pathogenesis (Chalasani et al., 2012). About 15%–25% of individuals with NAFLD progress to non-alcoholic steatohepatitis (NASH), which is characterized by vesicular steatosis, hepatic lobular inflammation, hepatocellular lipid accumulation and ballooning, periportal hepatocellular fibrosis, and perisinusoidal fibrosis (Takahashi and Fukusato, 2014). Lipid-laden hepatocytes undergo oxidative stress and lipid peroxidation, releasing pro-fibrotic and pro-inflammatory mediators that activate hepatic stellate cells and recruit immune cells (Cai et al., 2005). Strong associations exist between NAFLD/NASH and obesity, dyslipidemia, type 2 diabetes, or metabolic syndrome (Marjot et al., 2020; Younossi et al., 2018; Targher et al., 2007). Given limited therapeutic options and an incompletely understood pathogenesis, lifestyle interventions-including dietary modification, exercise, and weight reduction-remain the cornerstone of management (Moore et al., 2020). Regular exercise reduces hepatic fat and enhances insulin sensitivity, thereby mitigating NASH progression (Wong et al., 2013; Vilar-Gomez et al., 2015; Winn et al., 2018).

Recently, there has been an intensive attempt to study dietary elements that aid in the prevention and treatment of NAFLD (Yan et al., 2020; Lei et al., 2019; Wei et al., 2023; Choi et al., 2023). Garlic (*Allium sativum* L.), a perennial herb *Angiosperms* of the genus *Allium* (family *Amaryllidaceae*), is one of the most ancient cultivated plants, has been utilized for both culinary and medical purposes owing to its medicinal properties (Xiao et al., 2013a; Shin et al., 2014; Wu et al., 2015). Empirical research has demonstrated that garlic possesses the capacity to resolve metabolic disorders by enhancing glucose metabolism and lipid distribution. Additionally, the ingredients of garlic have the ability to ameliorate NAFLD and exhibit various biological effects, including reducing oxidative stress, antihyperlipidemic, antibacterial, anti-obesity, hepatoprotective and anti-cancer (Zhang et al., 2017; Ankri and Mirelman, 1999; Ernst, 1997). Preclinical and clinical studies have confirmed the therapeutic potential of garlic as a therapeutic agent for a variety of diseases, such as atherosclerosis (Karagodi et al., 2016), NAFLD (Sangouni et al., 2020) and prevention of viral infections (Rouf et al., 2020). The main bioactive components in garlic are organosulfur compounds, representative of which are: Allicin, Alliin, S-allyl-cysteine (SAC), and γ-glutamyl-S-allyl-cysteine (GSAC) (Shang et al., 2019). Allicin, the most potent compound in garlic with a strong garlic flavor and plays an important role in the treatment of metabolic liver diseases such as obesity and metabolic disorders (Zhan et al., 2020). SAC, another major bioactive compound in garlic, is converted from GSAC and can be converted to Alliin (Utama et al., 2024). Hwang et al. identified SAC as a non-toxic natural product in garlic, exhibits diverse pharmacological properties, including antioxidant and hepatoprotective effects (Hwang et al., 2013). Hence, garlic possesses significant potential as a therapeutic agent for the prevention of NAFLD.

Ferroptosis is a newly recognized type of cell death that, in contrast to other programmed cell death pathways, results in the generation of iron-dependent lipid peroxidation (Jiang et al., 2021). This form of cell death is triggered by the disturbance of internal metabolic pathways, resulting in a substantial buildup of lipid peroxides (Abdel Halim et al., 2024). It is intricately linked to the regulation of iron within the cell and the maintenance of lipid homeostasis (Stockwe et al., 2017). The fundamental processes underlying ferroptosis primarily involve an imbalance in the amino acid antioxidant system, lipid peroxide accumulation, and disruptions in iron metabolism. Among these, the accumulation of lipid peroxides (LPO) serves as the core mechanism of ferroptosis (Yang and Stockwell, 2016). Ferroptosis inducers like erastin exert their effects on glutathione peroxidase through many mechanisms, resulting in a decrease in cellular antioxidant capacity, ROS accumulation, and ultimately oxidative cell death (Xie et al., 2016). Significantly, NAFLD patients with higher levels of circulating iron (defined as more than 1.5 times the upper limit of normal) were found to have a strong association with hepatic iron deposition and tissue inflammation. It was also concluded that elevated iron levels in the blood are an independent risk factor for the progression of NAFLD to NASH and liver fibrosis (Kowdley et al., 2012). Recent studies suggest that ferroptosis is linked to inflammatory episodes of steatohepatitis in the early stages of NASH in mouse models (Tsurusaki et al., 2019). Furthermore, there is compelling evidence indicating that ferroptosis and disruptions in lipid metabolism play a crucial role in the progression of NASH (Li XY. et al., 2020). Thus, interfering with ferroptosis signaling in the liver is emerging as a novel and prospective therapeutic strategy for NASH.

Ultrafine powders are powder materials with particle sizes controlled to be within 10 µm. These powders have garnered more attention compared to conventional powders by virtue of their extensive surface area and enhanced reactivity (Li Y. et al., 2020; Li et al., 2022). The physical and chemical properties of solids undergo substantial changes when they are reduced to micro- or even nano-scale sizes, and exhibit significant alterations in their optical, electrical, magnetic, mechanical, thermodynamic, surface, and interface properties (Muttakin et al., 2015). These changes give rise to a multitude of distinctive characteristics, offering extensive potential applications in catalysis, light filtering, light absorption, medicine, magnetic media, and new materials. An important benefit in the pharmaceutical industry is its ability to improve the absorption of drugs and produce the necessary therapeutic effects using lower doses of expensive and limited herbs (Li Y. et al., 2020; Wang et al., 2023; Zhao et al., 2009). The ultrafine garlic powder (UGP) used in this study is a processed formulation rather than an approved pharmaceutical product; its clinical indications and safety profile remain unestablished, and comprehensive toxicological evaluations are necessary to support potential clinical translation.

In this study, we applied high-speed and energy particle collision equipment to produce UGP, and as compared with TGP, UGP possess a smaller particle size and contact angle, along with increased solubility, dispersibility, and antioxidant capacity. In addition, GC-MS/LC-MS analysis confirmed that the main bioactive components in garlic, including allicin, allicin, SAC and GSAC, were present at higher levels in UGP than in TGP. In our *in vitro* experiments, we used palmitic acid-laden THLE2 to investigate the beneficial role of UGP in lipid ROS accumulation and mitochondrial function, in comparison to TGP. On the other hand, we stimulated THLE2 cells with erastin to induce ferroptosis in hepatocytes and noticed that UGP significantly reduced cell death, ROS production, and mitochondrial dysfunction, as compared with TGP. For *in vivo* approaches, we established 2 mouse models of NASH and observed that UGP showed significant therapeutic benefits in interfering with the development of steatosis, hepatocyte ferroptosis, inflammation, and fibrosis. Our findings highlight the protective effects of garlic in the development and progression of NASH through inhibiting hepatocyte lipotoxicity and ferroptosis, shedding new insights into the application of ultrafine powder technology in the development of natural herbal medicine.

## 2 Materials and methods

### 2.1 Chemicals and antibodies

Chemicals used in this study: Bovine Serum Albumin (BSA, BS114-100g, Biosharp, Hefei, Anhui, China), 4′,6-diamidino-2-phenylindole (DAPI, D1306, Thermo Fisher Scientific, Waltham, Massachusetts, USA), Erastin (E424821, Aladdin, Shanghai, China), Ferrostatin-1 (Fer-1, F408509, Aladdin, Shanghai, China), Palmitic acid (P815431, Macklin, Shanghai, China), Paraformaldehyde (PFA, 28908; Thermo Fisher Scientific, Waltham, Massachusetts, USA), Triton X-100 (T8787, Sigma,St. Louis, Missouri, USA).

Antibodies used in this study: ACTA2 (ab124964, Abcam, Cambridge, United Kingdom), COL1A1 (ab138492, Abcam, Cambridge, United Kingdom), phospho-ERK1/2 (4370, CST,Danvers, Massachusetts, USA), ERK1/2 (4695, CST,Danvers, Massachusetts, USA), FN (fibronectin, ab268020, Abcam, Cambridge, United Kingdom), GAPDH (2118, CST,Danvers, Massachusetts, USA), rabbit Alexa Fluor 488 secondary antibody (ab150077, Abcam), rabbit HRP (7074, CST,Danvers, Massachusetts, USA).

### 2.2 Preparation and validation of UGP and TGP

TGP was purchased from VEpiaopiao (Production Number: SC11735062200735). UGP was supplied by MATIFE BIO Ltd., and the garlic used was of the same variety and origin as TGP. Briefly, a high-speed and energy particle collision equipment was used to produce UGP, and the residual moisture in the garlic powder was eliminated by temperature and pressure control, and finally the micro- and nano-powders with smaller sizes were obtained in the organizer. The particle sizes of UGP and TGP were analyzed using an ultra-high-speed smart particle size analyzer (MASTER SIZER 3000, Malvern Instruments Ltd., UK). The morphology of UGP and TGP was studied using scanning electron microscopy (SEM, FEI, Czech Republic). A multifunctional imaging electron spectrometer (XPS, Thermo ESCALAB 250XI, Thermo Fisher Scientific, USA) was used to determine the composition of UGP and TGP. A Fourier Transform Infrared Spectrometer (FT-IR, Nicolet iS5, Thermo Nicolet Corporation, USA) was used to identify functional groups in UGP and TGP. The hydrophilicity of UGP and TGP was investigated using Optical Contact Angle Meter (Theta Flex, Biolin, Sweden). The antioxidant capacity of UGP and TGP was assessed using the DPPH Free Radical Scavenging Capacity Assay Kit (BC4750, Solarbio, Beijing, China), Superoxide Anion Activity Content Assay Kit (BC1290, Solarbio, Beijing, China), and the Hydroxyl Free Radical Scavenging Capacity Assay Kit (BC1320, Solarbio, Beijing, China).

### 2.3 GC-MS/LC-MS analysis of UGP and TGP

100 mg of the UGP or TGP sample was mixed with 1 mL of dichloromethane in an EP tube, sonicated for 10 min, mixed well and centrifuged, the supernatant was collected through an organic filter membrane. The content of Allicin in UGP and TGP was determined by gas chromatography-mass spectrometry (GC-MS, Xevo TQ-GC, Waters). The separation was conducted on a SUPELCO Discovery DB-5MS column (30 m × 0.25 mm ID) with split ratio of 5:1 and sample volume of 2 µL. Helium was used as the carrier gas at a flow rate of 1 mL/min, and the temperature of the injection port was 260 °C. The heating program was as follows: the initial temperature was 50 °C, held for 2 min, then the temperature was continuously increased to 110 °C at a rate of 10 °C/min, held for 1 min, and then the temperature was continuously increased to 260 °C at a rate of 20 °C/min, held for 0 min, for a total of 16.5 min. MS conditions: ionization was in EI mode, SIR mode was selected for the analysis, scanner ions are 81 and 41, and solvent delaied for 3 min. On the other hand, Alliin, SAC and GSAC were determined by liquid chromatography-quadrupole orbit trap mass spectrometry (LC-MS, Q-Exactive Focus, Thermo Fisher Scientific, Waltham, Massachusetts, USA). The separation was conducted on a ACQUIRE UPLC BEH C18 column (1.7 um, 2.1 × 100 mm) using a binary mobile phase composed of A (water supplemented with 0.1% formic acid) and B (acetonitrile). MS conditions: SIM Scanning Mode, ms2:17500, collision energy: 35, AGC: 2e5, maximum IT: 100 ms; isolation window: 4.0 m/z. The monitored mass numbers (m/z values) of Alliin, SAC and GSAC were 178.05200, 162.05700 and 291.0900, respectively.

### 2.4 Cell culture

Human normal hepatocytes (THLE2) and hepatic stellate cells (HSCs) (LX-2) were purchased from the American Type Culture Collection (ATCC) and cultured in BEGM kit medium and RPMI medium supplemented with 10% FBS, respectively. Cells were stimulated with palmitate (0.5 mM), erastin (10 μM), Fer-1 (5 mM), UGP (10 mg/mL), or TGP (10 mg/mL) for 24 h as outlined in the main text and/or figure legends.

### 2.5 Flow cytometry

Cell death analysis was performed by staining cells with Dead Cell Apoptosis Kit with Annexin V FITC and PI (CA1020; Beyotime, Shanghai, China). Lipid Peroxidation was examined by staining cells with Liperfluo dye (L248; Dojindo, Kumamoto, Japan). Cellular Fe^2+^ levels were examined by staining cells with FerroOrange dye (F374; Dojindo, Kumamoto, Japan). Cells were then quantified with the flow cytometer (Beckman, Brea, California, USA) and analyzed with CtyExpert software: the preliminary FSC/SSC gates of the starting cell population were 10,000 events. Debris (SSC-A vs. FSC-A) and doublets (FSC-H vs. FSC-A) were excluded. Boundaries between “positive” and “negative” staining were set at 10^4^ (Ng et al., 2019).

### 2.6 ROS detection

Cells were seeded on 24-well plate (6 × 10^3^ cells/well). Twenty-four hours following stimulation of palmitate, erastin, Fer-1, UGP, or TGP, cells were washed, incubated with 10 µM of DCFDA solution (S0033, Beyotime, Shanghai, China) for 45 min at 37 °C in the dark, and rinsed with the dilution buffer according to the manufacturer’s protocol. Live cells with positive DCF staining were imaged with a filter set appropriate for fluorescein (FITC) using a fluorescence microscope (Nikon, Tokyo, Japan) (Lyu et al., 2024).

### 2.7 Mitochondrial membrane potential detection

Cells were seeded on 24-well plate (6 × 10^3^ cells/well). Twenty-four hours following stimulation of palmitate, erastin, Fer-1, UGP, or TGP, cells were washed, incubated with 200 nM of Mito-Tracker Red CMXRos solution (C-1049B; Beyotime, Shanghai, China) for 30 min at 37 °C in the dark, and rinsed with the dilution buffer according to the manufacturer’s protocol. Mitochondrial membrane potential with positive Mito-Tracker staining were imaged with a filter set appropriate for PE using a fluorescence microscope (Nikon, Tokyo, Japan) (Dong et al., 2023).

### 2.8 Immunofluorescence (IF)

LX-2 cells were seeded on 24-well plate (6 × 10^3^ cells/well) and incubated with conditional medium for 24 h before staining. Cells were fixed in 4% PFA for 20 min, washed with PBS, and non-specific sites were blocked with 5% BSA in PBS for 2 h. Cells were incubated with COL1A1 or ACTA2 antibody overnight (4 °C), followed by incubation with the appropriate Alexa Fluor 488 secondary antibody for 1 h (RT) and DAPI for 10 min. Positive COL1A1 or ACTA2 staining were imaged with a filter set appropriate for fluorescein (FITC) using a fluorescence microscope (Nikon, Tokyo, Japan) (Ng et al., 2019).

### 2.9 Experimental animals and housing conditions

The study was conducted in mice because murine models have well-established pathophysiological relevance to human NASH and allow mechanistic exploration of ferroptosis-related injury. Male mice were used to minimize hormonal variability associated with the estrous cycle, which may affect hepatic lipid metabolism and oxidative stress responses. Animal experiments were approved by the Animal Ethics Committee of the Tianjin University Laboratory Animal Center (Tianjin, China) (Approval No. TJUE-2024–127). All procedures complied with national and institutional animal care guidelines. Male C57BL/6J mice (6–8 weeks, 22–25 g; Beijing Vition Technology Co., Ltd. for Laboratory Animal Services) were acclimated for ≥7 days and housed 5 per cage in individually ventilated polycarbonate cages with standard bedding, enrichment, 12 h light/dark cycle, 22 °C ± 2 °C, and 50% ± 10% humidity. Standard chow and water were provided *ad libitum*; experimental groups received specific diets (high-fat or choline-deficient high-fat). Painful procedures were performed under anesthesia (isoflurane), and euthanasia followed approved methods (e.g., CO_2_), with predefined humane endpoints. Animals were randomly assigned to groups, and all analyses were performed by investigators blinded to group assignment.

### 2.10 Study design and experimental groups

In this study, 60 C57BL/6N male mice (6–8 weeks old) were randomly assigned to different experimental groups, with 6 mice per group. The groups were as follows:

Acute Liver Fibrosis Model (CDHF): Mice were fed a methionine- and choline-deficient diet supplemented with 60 kcal% fat (CDHF, A06071301B, Research Diets) for 4 weeks. The control group was fed normal chow (NC, Specialty Feeds). Treatment regimens for the groups were as follows (Jiang et al., 2022): NC (normal chow), CDHF (acute liver fibrosis model), CDHF + Fer-1: 2.5 μmol/kg via intraperitoneal injection (IP) 3 times per week for 4 weeks, CDHF + UGP: 500 mg/kg administered orally (oral) 3 times per week for 4 weeks, CDHF + TGP: 500 mg/kg administered orally (oral) 3 times per week for 4 weeks.

Chronic Liver Fibrosis Model (HFD): Mice were fed a rodent diet with 60 kcal% fat (HFD, D12492, Research Diets) for 12 weeks. The control group was fed normal chow (NC, Specialty Feeds). Treatment regimens for the groups were as follows (Jiang et al., 2022): NC (normal chow), HFD (chronic liver fibrosis model), HFD + Fer-1: 2.5 μmol/kg via intraperitoneal injection (IP) 3 times per week for 12 weeks, HFD + UGP: 500 mg/kg administered orally (oral) 3 times per week for 12 weeks, HFD + TGP: 500 mg/kg administered orally (oral) 3 times per week for 12 weeks.

At the end of each treatment period, blood and liver tissues were collected for subsequent analyses.

### 2.11 Colorimetric assays

Cell viability and LDH activity were quantified using CCK8 Activity Assay Kit (CA1210, Solarbio, Beijing, China) and LDH activity Assay Kit (BC0685, Solarbio, Beijing, China). Alanine transaminase (ALT) activity in cell culture supernatants and mouse serum was determined using ALT/GPT Assay Kit (C009-2-1, Njjcbio, Nanjing, Jiangsu Province, China). Liver Collagen, GSH, triglyceride and cholesterol levels were measured using HYP Assay Kit (A03021, Njjcbio, Nanjing, Jiangsu Province, China), GSH Assay Kit (A00621, Njjcbio, Nanjing, Jiangsu Province, China), Triglyceride Content Assay Kit (A110-1-1, Njjcbio, Nanjing, Jiangsu Province, China) and Total Cholesterol Assay Kit (BC1980, Solarbio, Beijing, China), respectively. Serum levels of aspartate aminotransferase (AST), iron, malondialdehyde (MDA) and superoxide dismutase (SOD) were evaluated using AST/GOT Kit (C010-2-1, Njjcbio, Nanjing, Jiangsu Province, China), Serum Iron (Fe^3+^+Fe^2+^) Assay Kit (A039-1-1, Njjcbio, Nanjing, Jiangsu Province, China) and SOD Assay Kit (A001-3, Njjcbio, Nanjing, Jiangsu Province, China), respectively. Liver iron (Fe^3+^+Fe^2+^) and LPO levels were quantified using Tissue Iron (Fe^3+^+Fe^2+^) Assay Kit (A039-2-1, Njjcbio, Nanjing, Jiangsu Province, China) and LPO Assay kit (A106-1, Njjcbio, Nanjing, Jiangsu Province, China), respectively. All colourimetric assays were performed according to the manufacturer’s protocol.

### 2.12 Liver tissue processing and histological analysis

Mouse livers were fixed in 4% PFA, embedded in paraffin, and cut into 6 µm-thick slices. Sections were stained with hematoxylin and eosin (H&E), Masson’s Trichrome (MT), or Prussian blue (PB) staining according to standard protocol and examined by light microscopy (Nikon, Tokyo, Japan). For each animal, ten representative microscopic fields were captured for each section under the same magnification and exposure conditions.

### 2.13 RT-qPCR

The RNAeasy™ Animal RNA Isolation Kit with Spin Column (R0027, Beyotime, Shanghai, China) was used to extract total RNA from snap-frozen liver tissues. PCR amplifications were performed using cDNA Synthesis Master Mix (D7185, Beyotime, Shanghai, China). Gene expression was analyzed in duplicate by SYBR green (D7260, Beyotime, Shanghai, China) technology using CFX96 (Bio-rad,Hercules, California, United States) over 40 cycles. Expression data were normalized to Gapdh mRNA expression and fold change was calculated using 2^−ΔΔCT^ method (Dong et al., 2021). The primer sequences are listed in Table 1.

**TABLE 1:** Primer sequences for RT-qPCR.

Gene	Forward primer sequence (5′-3′)	Reverse primer sequence (3′-5′)
Ms *Tnfα*	ATG​AGA​AGT​TCC​CAA​ATG​GC	CTC​CAC​TTG​GTG​GTT​TGC​TA
Ms *Il1β*	TGC​CAC​CTT​TTG​ACA​GTG​ATG	ATG​TGC​TGC​TGC​GAG​ATT​TG
Ms *Il6*	CTC​TGG​GAA​ATC​GTG​GAA​AT	CCA​GTT​TGG​TAG​CAT​CCA​TC
Ms *Il8*	ACCCGCTCGCTTCTCTGT	AAG​GGA​GCT​TCA​GGG​TCA​AG
Ms *Col1a1*	GGG​GCA​AGA​CAG​TCA​TCG​AA	GTC​CGA​ATT​CCT​GGT​CTG​GG
Ms *Col3a1*	GTC​CAG​GGA​TAC​GGG​GTA​TG	GAG​CAC​CGA​CTT​CAC​CCT​TT
Ms *Acta2*	TGG​AGA​AGC​CCA​GCC​AGT​CG	CCA​GCG​AAG​CCG​GCC​TTA​CA
Ms *Fn1*	ATA​AGC​CTC​TGC​TCT​TGG​GG	CTC​ACA​CCC​TGG​GCT​CCT​TT
Ms *Acsl4*	GCA​CCT​TCG​ACT​CAG​ATC​ACA	GAA​GCC​AGC​AAT​AAA​GTA​CAC​AGA
Ms *Ncoa4*	TAT​CCA​GGT​GCC​AGA​GCA​GA	GGC​ATC​GCT​GAA​GAA​ACT​GC
Ms *Gpx4*	CCG​TCT​GAG​CCG​CTT​ACT​TA	GTG​ACG​ATG​CAC​ACG​AAA​CC
Ms *Slc7a11*	AAT​ACG​GAG​CCT​TCC​ACG​AG	CTC​CAG​GGG​CAG​TCA​GTT​AG
Ms *Fsp1*	ACT​TCC​TCT​CTC​TTG​GTC​TGG​T	ACT​TGT​CAC​CCT​CTT​TGC​CT
Ms *Gapdh*	CTG​GAA​AGC​TGT​GGC​GTG​AT	GAC​GGA​CAC​ATT​GGG​GGT​AG

### 2.14 Immunoblotting

Mouse liver samples were lysed using a 100:1 mixture of RIPA buffer and PMSF from (R0020,Solarbio, Beijing, China), followed by quantification of each protein concentration using the BCA Protein Assay Kit (PC0020, Solarbio, Beijing, China). The protein samples were separated via SDS-PAGE and transferred to PVDF membranes. These membranes were covered with 5% skimmed milk powder, left overnight at 4 °C with a primary antibody and washed with 1% Tween 20 in TBS afterwards. Anti-rabbit HRP secondary antibody was used to incubate the membranes for 2 hours at room temperature before visualizing the protein bands using the ECL detection system (Dong et al., 2021).

### 2.15 RNA-seq analysis

The RNAeasy™ Animal RNA Isolation Kit with Spin Column (R0027, Beyotime, Shanghai, China) was used to extract total RNA from snap-frozen liver tissues. The Agilent Bioanalyzer 2100 system (Agilent Technologies, CA, USA) served to evaluate library quality and RNA integrity using the RNA Nano 6000 Assay Kit. Following cluster creation, 150 bp paired-end reads were produced from the library preparations sequencing using an Illumina Novaseq platform. Using Hisat2 (v2.0.5, accessed on 1 March 2025), an index of the reference genome was constructed, and paired-end clean reads were aligned to the reference genome. Using a reference-based methodology, the mapped reads of every sample were constructed using StringTie (v1.3.3b, accessed on 1 March 2025) (Pertea et al., 2015). The read counts that were mapped to each gene were counted using FeatureCounts (v1.5.0-p3, accessed on 1 March 2025). Next, each gene’s FPKM was computed using its length and the number of reads that were mapped to it. The DESeq2 R package (v1.20.0, accessed on 1 March 2025) was utilized to identify differentially expressed genes (DEGs) based on a corrected p value of less than 0.05. Gene Ontology (GO) enrichment analysis of DEGs was conducted using the clusterProfiler R package (v3.8.1, accessed on 1 March 2025), with correction for gene length bias. GO terms with adjusted p-values <0.05 were deemed significantly enriched. KEGG pathway enrichment analysis was also performed using clusterProfiler, while gene set enrichment analysis (GSEA) was conducted with the local GSEA software tool (v4.3.2, Broad Institute, accessed on 1 March 2025), applying GO and KEGG datasets independently.

### 2.16 Statistical analysis

All statistical analyses were conducted using GraphPad Prism software (version 10.0). Normal distribution was confirmed by the Kolmogorov-Smirnov test. Simple two-tailed Student’s t-tests were used for experimental setups comparing two conditions. For comparisons between more than two conditions, one-way ANOVA with Tukey’s correction (when several conditions were compared to each other) were used. The criterion for statistical significance was set at *P* < 0.05. We have ensured that all relevant intergroup comparisons were considered and reported in the Statistical Analysis section.

## 3 Results

### 3.1 Characterization of UGP and TGP

**FIGURE 1:**
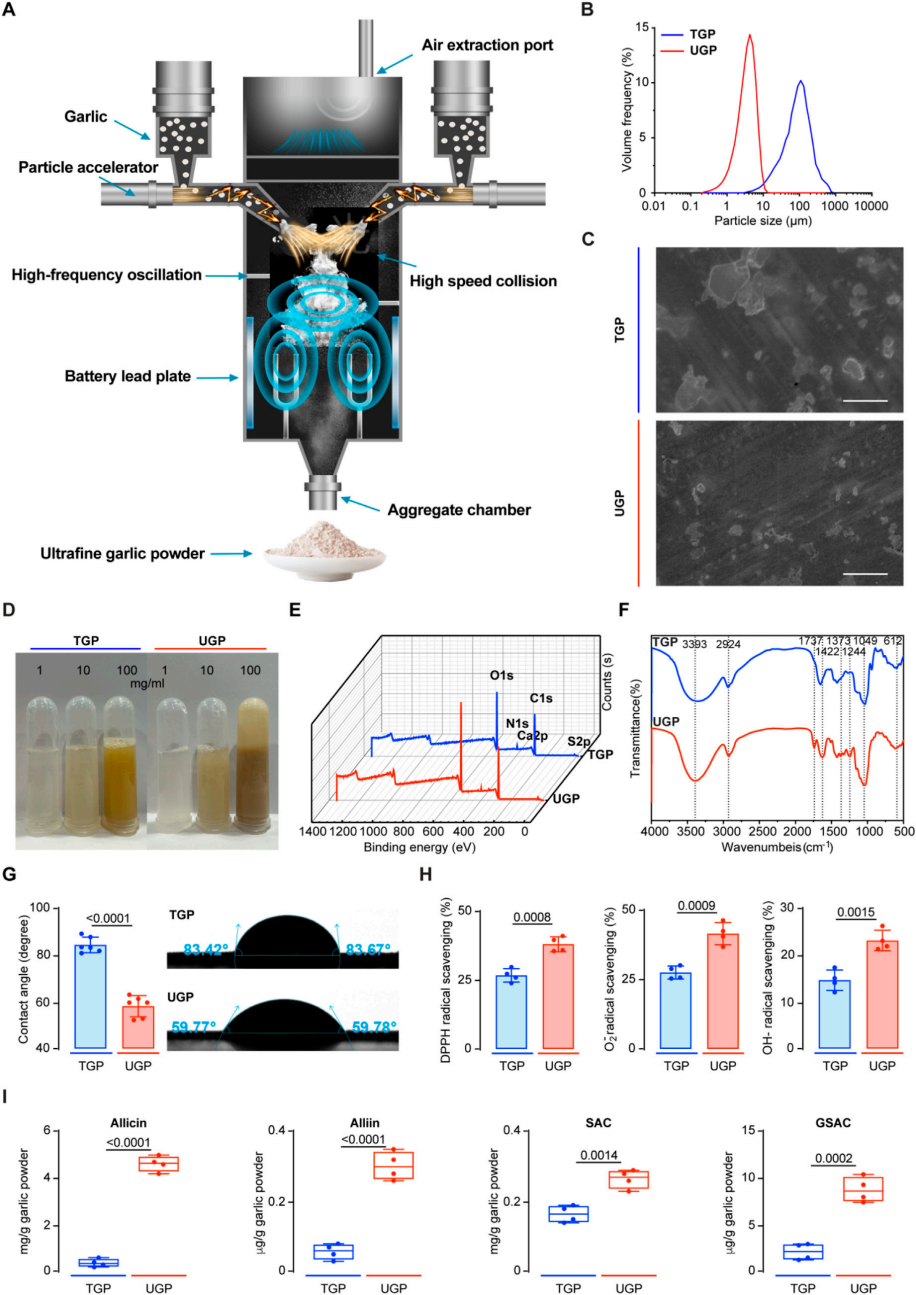
Characterization of TGP and UGP. **(A)** Schematic of the production of UGP by a high-speed and energy particle collision equipment. **(B)** Particle size distribution of TGP and UGP. **(C)** SEM images of TGP and UGP (scale bar = 100 μm). **(D)** Solubility and dispersibility of TGP and UGP in H_2_O. **(E)** XPS spectra of TGP and UGP. **(F)** FT-IR spectra of TGP and UGP. **(G)** Contact angle measurement of TGP and UGP (n = 6). **(H)** Antioxidant capacity (DPPH radical scavenging, O_2_^−^ radical scavenging, and OH^−^ radical scavenging) analyses of TGP and UGP (n = 4). (TGP: Traditional garlic powder; UGP: Ultrafine garlic powder.).

**FIGURE 2:**
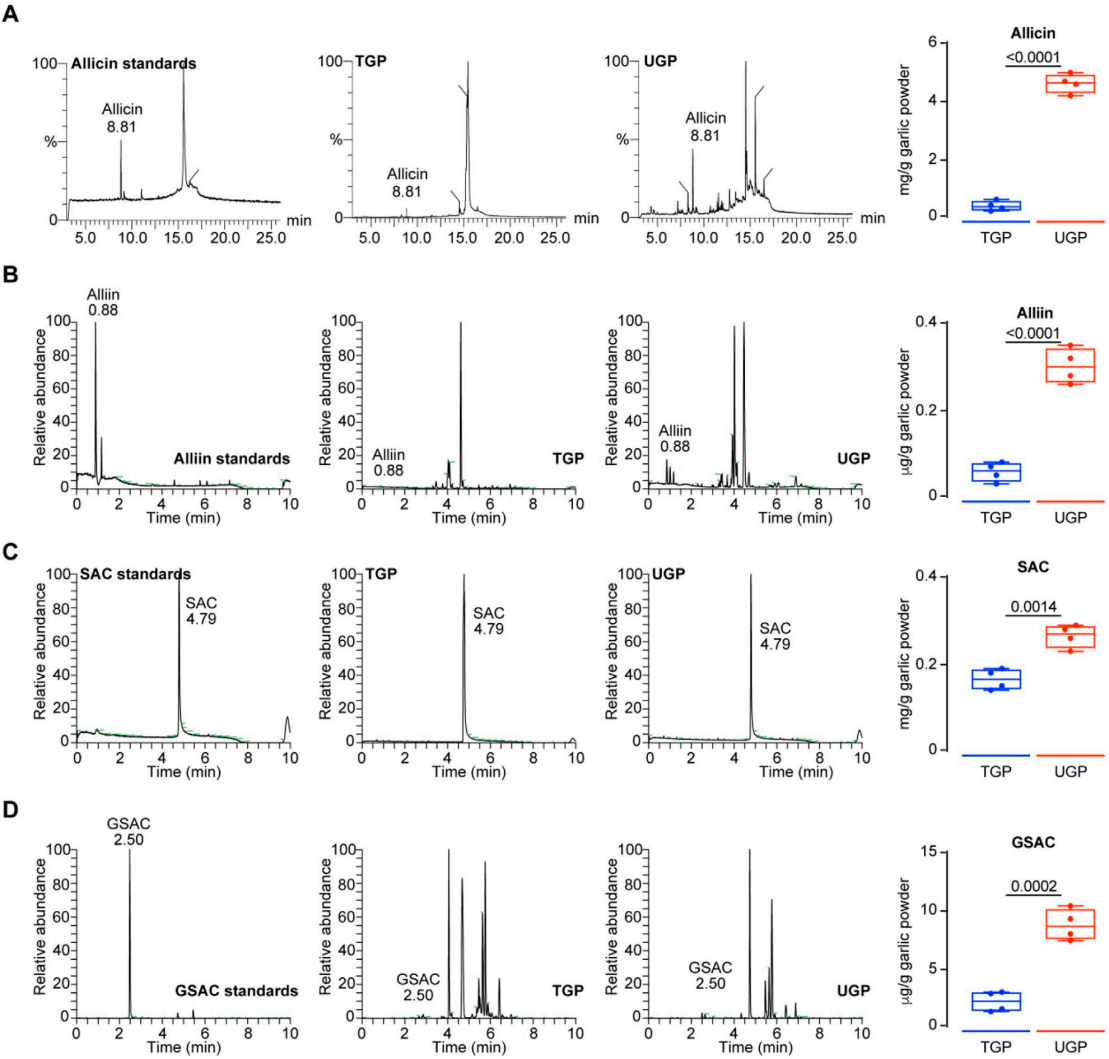
SRM chromatograms and quantification of four standard compounds including Allicin **(A)** Alliin **(B)** SAC **(C)** and GSAC **(D)** in TGP and UGP (n = 4), respectively. (GSAC: γ-Garlic S-allyl cysteine; SAC: S-allyl cysteine; TGP: Traditional garlic powder; UGP: Ultrafine garlic powder).

UGP was produced by a high-speed and energy particle collision equipment (Figure 1A). We first compared the particle sizes of UGP and TGP, and d (0.5) for UGP was approximately 4 μm, which was more than 30 times smaller than that of TGP (Figure 1B). Additionally, SEM images displayed a marked reduction in particle size in UGP, as compared with TGP. The particle angles became indiscernible, and the borders became less distinct for UGP (Figure 1C). UGP is more soluble and dispersed in H_2_O, PBS and DMSO than TGP in a dose-dependent manner (Figure 1D; Supplementary Figure S1). XPS assay was conducted to confirm that both UGP and TGP belonged to the same species possessing the same elements, as shown in Figure 1E. Furthermore, FT-IR demonstrated that UGP would exhibit hydroxyl functional groups, which would greatly enhance its biological and pharmacological effects (Figure 1F). The combined effects of reduced particle size and abundant surface hydroxyl groups increase the effective specific surface area and improve wettability, thereby promoting faster dissolution and greater molecular release in aqueous environments compared with TGP. These physicochemical modifications are expected to enhance interactions with biological interfaces and increase the bioavailable fraction of active compounds. The contact angle measurements suggest that both UGP and TGP exhibit hydrophilic properties. UGP has higher hydrophilicity compared to TGP, resulting in a decreased rate of drug loss (Figure 1G). The increased hydrophilicity and reduced contact angle of UGP enhance its dispersibility and minimize aggregation in physiological media, providing a physicochemical basis for more efficient cellular interaction and reduced extracellular sequestration. Upon evaluating the scavenging potential of UGP and TGP against DPPH, O_2_^−^, and OH^−^ radicals, it was observed that UGP exhibits a higher antioxidant capacity (DPPH radical scavenging, O_2_^−^ radical scavenging, and OH^−^ radical scavenging) compared to TGP (Figure 1H). The enhanced radical-scavenging activity of UGP aligns with its higher content of biologically active sulfur-containing compounds, suggesting an increased capacity to mediate redox-dependent biological effects in cellular systems. The GC-MS/LC-MS method was successfully used to quantify the four major bioactive components in TGP and UGP, with the four compounds in UGP being more abundant than those in TGP (Figure 1I). Despite the complexity of the garlic matrix, the high selectivity and sensitivity of the method effectively minimized the interference of non-target components, resulting in clear SRM peaks for allicin, allicin, SAC and GSAC at the expected retention times (Figure 2). Quantitative analyses (GC-MS and LC-MS) confirmed that key organosulfur constituents, including allicin and SAC, are enriched in UGP relative to TGP, providing direct evidence of compositional alterations underlying its enhanced bioactivity. Collectively, the reduced particle size, improved solubility and hydrophilicity, surface hydroxylation, and higher abundance of active organosulfur compounds offer a coherent mechanistic explanation for the superior therapeutic effects of UGP compared with TGP, as these features are expected to enhance dissolution, promote cellular adhesion and contact, and increase the local concentration of active molecules available for uptake.

### 3.2 UGP significantly ameliorates palmitate-induced lipotoxic hepatocyte injury

**FIGURE 3:**
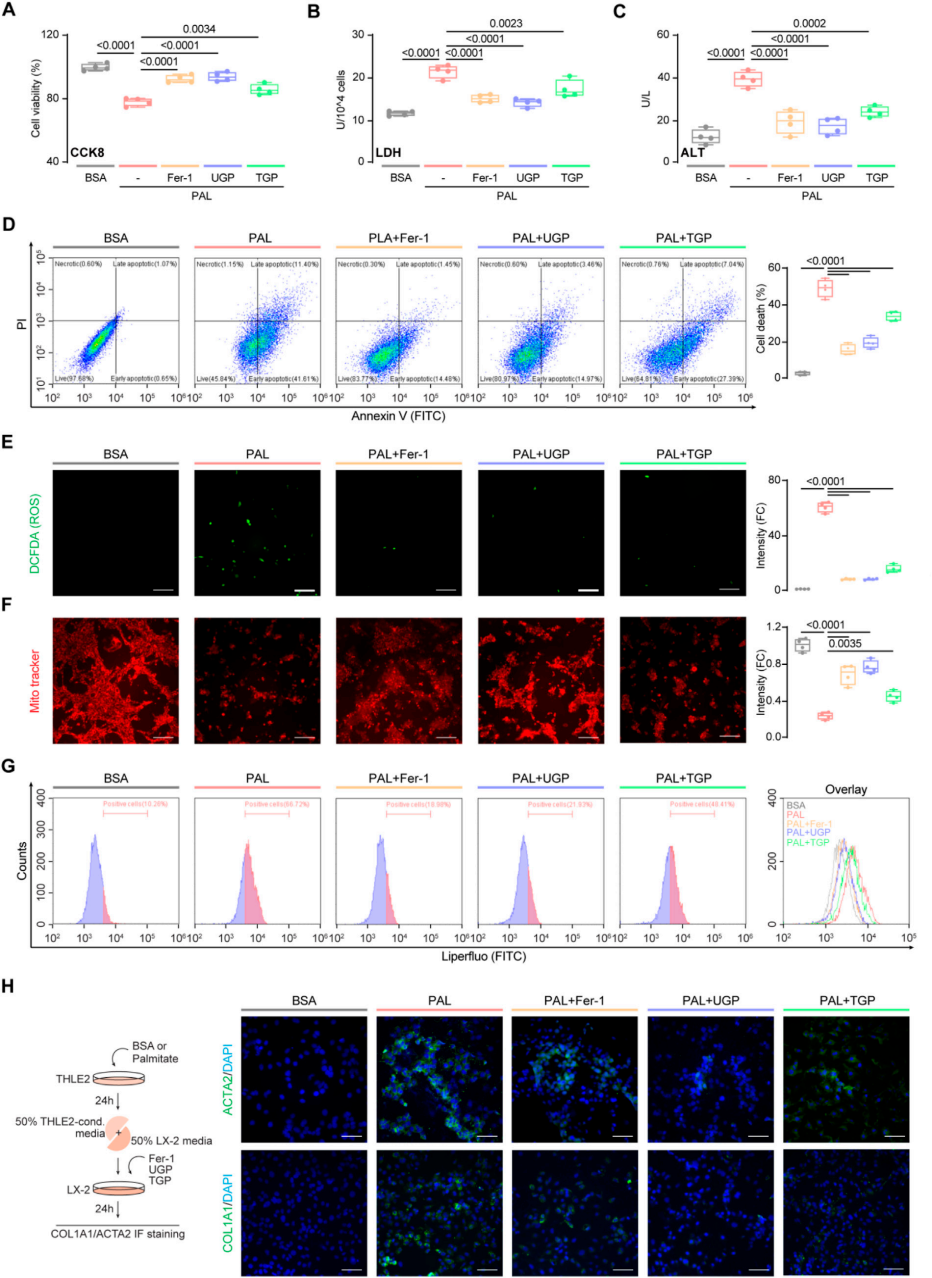
UGP ameliorates palmitatic acid-induced lipotoxic hepatocyte injury. Effects of palmitatic acid stimulation and Fer-1, UGP and TGP treatment of on human normal hepatocyte THLE2 cell viability was examined using CCK8 assay **(A)** and LDH assay **(B)** (n = 4). **(C)** Effects of palmitatic acid stimulation and Fer-1, UGP and TGP treatment of on THLE2 cell damage was determined by ALT assay (n = 4). **(D)** Cell death assay was performed using Annexin V/PI staining (n = 4). Annexin V^+^PI^−^, Annexin V^+^PI^−^, and Annexin V^+^PI^+^ cells were considered dead cells. Representative fluorescent images and quantification of DCFDA (2′,7′-dichlorofluorescein diacetate) **(E)** and Mito-Tracker Red CMXRos **(F)** staining for ROS and mitochondrial membrane potential detection (scale bar = 100 μm, n = 4). **(G)** Flow cytometry analysis of lipid peroxidation by Liperfluo staining on THLE2 cells. **(H)** Representative immunofluorescent images of ACTA2^+ve^ and COL1A1^+ve^ cells (scale bar = 100 μm). Data are shown as box-and whisker with median (middle line), 25th–75th percentiles (box), and min-max values (whiskers), one-way ANOVA with Tukey’s correction. (ALT: Alanine transaminase; BSA:Bovine serum albumin; Fer-1: Ferrostatin-1; PAL: Palmitic acid; ROS: Reactive oxygen species; TGP: Traditional garlic powder; UGP:Ultrafine garlic powder).

In order to investigate the role of UGP in fatty liver, we simulated hepatic lipotoxicity, a well-known initial factor in NAFLD that is linked to the production of pro-inflammatory and fibrosis markers (Friedman et al., 2018). To achieve this objective, we loaded normal human hepatocyte THLE2 cells with palmitate, a saturated fatty acid found elevated in the serum of NAFLD patients (Kleinfeld et al., 1996). UGP significantly improved palmitate-induced cell death, LDH activity, and ALT levels, as compared with TGP (Figures 3A–C). Annexin V-FITC/PI staining further confirmed that UGP significantly ameliorated palmitate-induced cell death in THLE2 cells (Figure 3D). In addition, palmitate-loaded THLE2 cells treated with UGP exhibited less ROS production and restored mitochondrial membrane potential, indicating a beneficial role of UGP in ROS-induced mitochondrial dysfunction and cell death as compared with TGP (Figures 3E,F). Flow analysis confirmed that UGP inhibited palmitate-induced lipid peroxidation, as evidenced by Liperfluo staining (Figure 3G). Human HSC LX-2 cells were exposed to conditioned media obtained from hepatocytes treated with either BSA control or palmitate. Our results demonstrate that media from lipotoxic hepatocytes significantly stimulated the expression of COL1A1 and ACTA2 in HSCs (Figure 3H). The addition of UGP to the conditioned media significantly blocked the expression of COL1A1 and ACTA2, in comparison to TGP (Figure 3H; Supplementary Figures S2A,B). These data demonstrated that UGP was able to ameliorate the lipotoxicity of hepatocytes and downstream activation of HSCs. Importantly, we employed Fer-1, an ferroptosis inhibitor, in our study and noted marked amelioration of the lipotoxic hepatocyte injury induced by palmitate. In addition, we have discovered that palmitate-loaded hepatocytes had significantly elevated levels of Fe^2+^, which were significantly reduced by UGP (Supplementary Figure S2C). All the above results indicated the potential role of ferroptosis in hepatocyte lipotoxicity.

### 3.3 UGP significantly ameliorates erastin-induced hepatocyte ferroptosis

**FIGURE 4:**
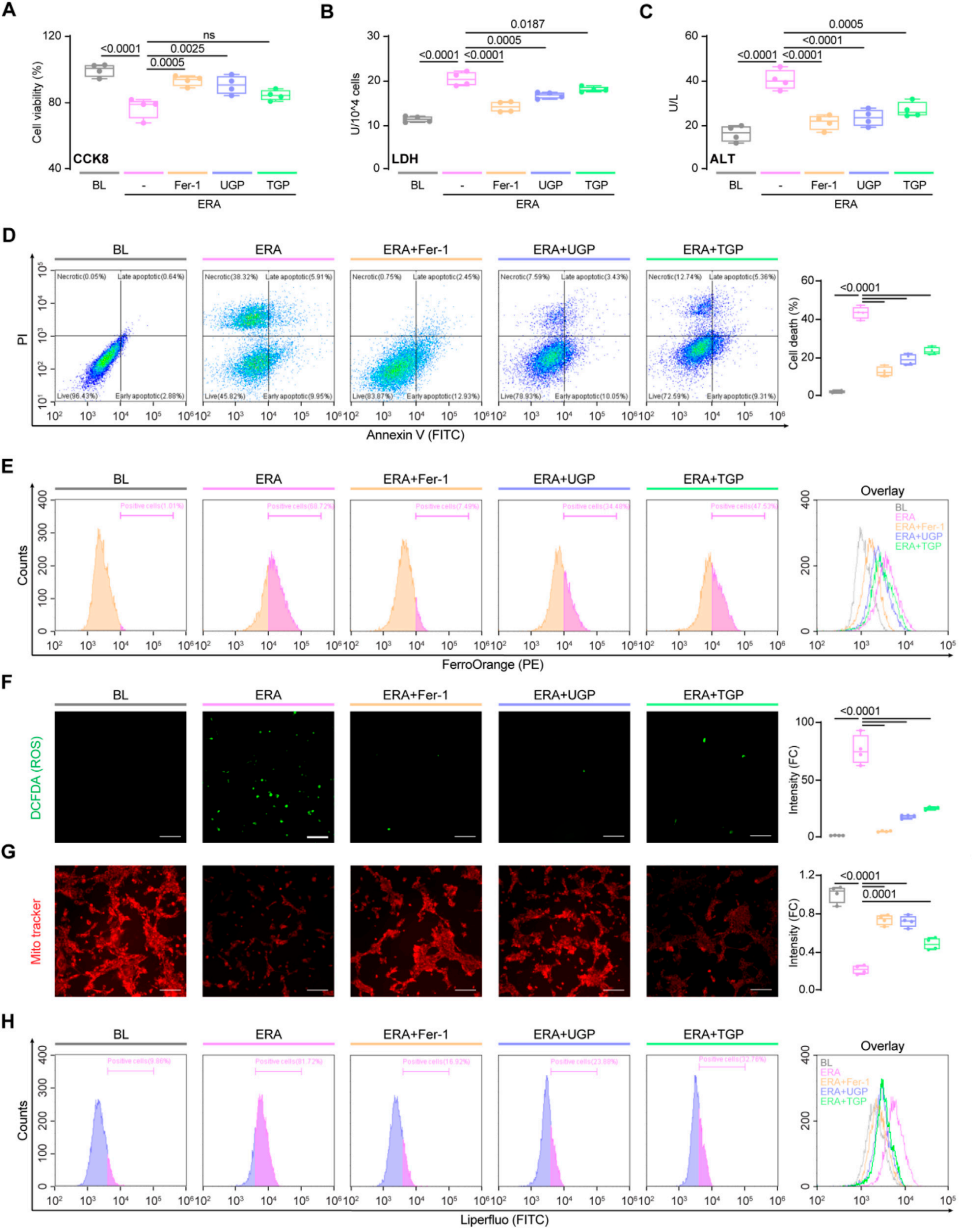
UGP significantly ameliorates erastin-induced hepatocyte ferroptosis. Effects of erastin stimulation and Fer-1, UGP and TGP treatment of on THLE2 cell viability was examined using CCK8 assay **(A)** and LDH assay **(B)** (n = 4). **(C)** Effects of erastin stimulation and Fer-1, UGP and TGP treatment of on THLE2 cell damage was determined by ALT assay (n = 4). **(D)** Cell death assay was performed using Annexin V/PI staining (n = 4). Annexin V^+^PI^−^, Annexin V^+^PI^−^, and Annexin V^+^PI^+^ cells were considered dead cells. **(E)** Flow cytometry analysis of cellular Fe^2+^ levels by FerroOrange staining on THLE2 cells. Representative fluorescent images and quantification of DCFDA (2′,7′-dichlorofluorescein diacetate) **(F)** and Mito-Tracker Red CMXRos **(G)** staining for ROS and mitochondrial membrane potential detection (scale bar = 100 μm, n = 4). **(H)** Flow cytometry analysis of lipid peroxidation by Liperfluo staining on THLE2 cells. Data are shown as box-and whisker with median (middle line), 25th-75th percentiles (box), and min-max values (whiskers), one-way ANOVA with Tukey’s correction. (ALT: Alanine transaminase; ERA: Erastin; Fer-1: Ferrostatin-1; ROS: Reactive oxygen species; TGP: Traditional garlic powder; UGP: Ultrafine garlic powder).

Subsequently, we instigated the process of hepatocyte ferroptosis by employing erastin, a compound that induces ferroptosis. After being exposed to erastin, there was a notable rise in cytotoxicity and hepatocyte damage (Figures 4A–C). To examine the potential inhibition effects of garlic powder on ferroptosis, cells were treated with UGP and TGP, whereas Fer-1 was used as a positive control for the inhibition. UGP significantly improved erastin-induced cell death, LDH activity, and ALT release, as compared with TGP (Figures 4A–D). FerroOrange is a small-molecule probe that is specific for measuring cellular Fe^2+^ levels, and by using FACS analysis, we confirmed that erastin-induced Fe^2+^ levels in hepatocytes were significantly elevated, which were further reduced by garlic powder (Figure 4E). ROS production results in GSH depletion and disruption of mitochondrial membrane structure, which respond to the degree of cellular damage. As compared with TGP, UGP significantly reduced ROS production, elevated mitochondrial membrane potential, and inhibited lipid peroxidation in erastin-stimulated THLE2 cells by fluorescent microscopy and flow cytometry examinations (Figures 4F–H). These data suggest the potential role of garlic powder in regulating ferroptosis signaling, which requires further study.

### 3.4 UGP protects against liver inflammation and fibrosis in CDHF-induced acute liver injury model

**FIGURE 5:**
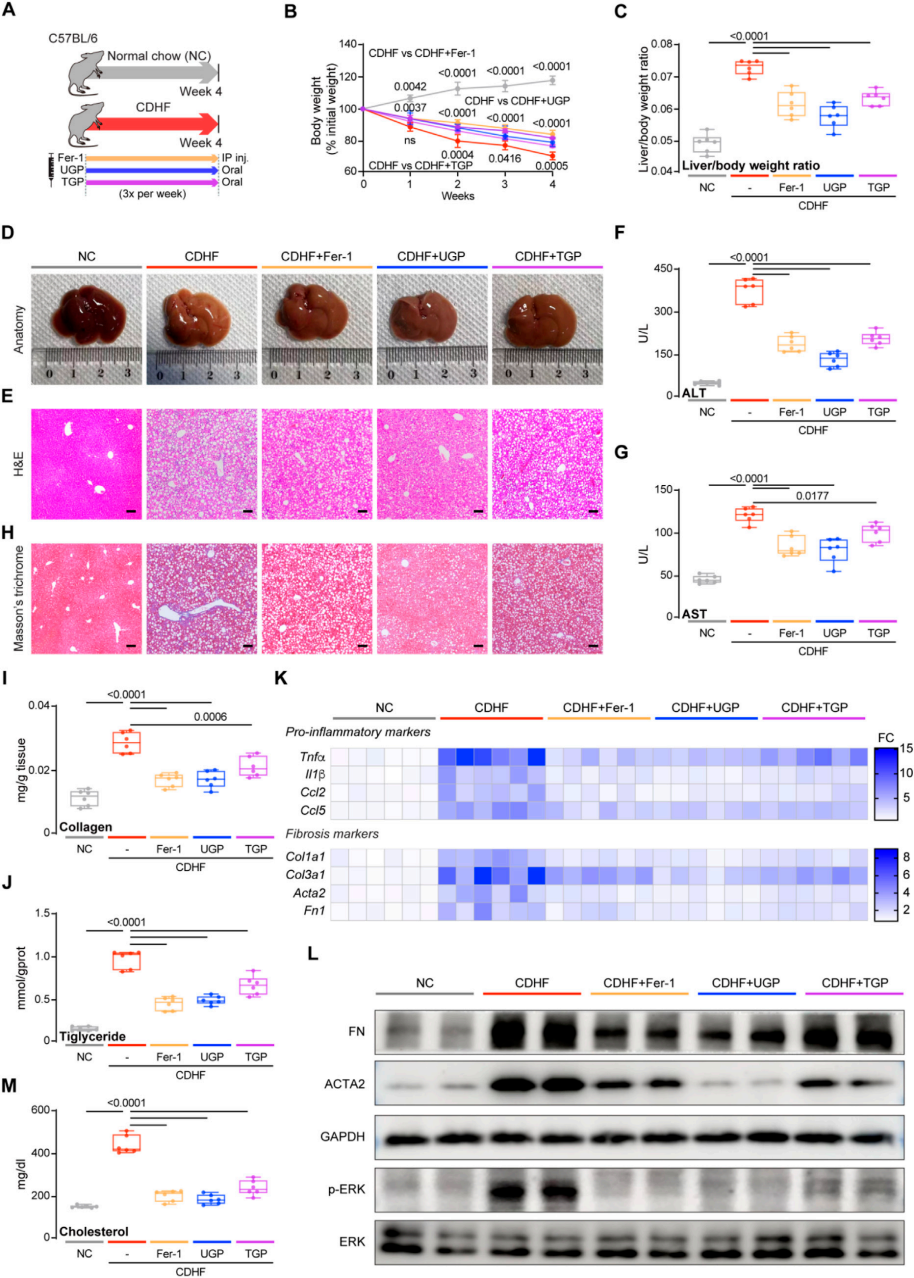
UGP protects against liver inflammation and fibrosis in CDHF-induced acute liver injury model. **(A)** Schematic of CDHF feeding in mice treated with Fer-1, UGP and TGP. **(B)** Mice body weight (shown as a percentage (%) of initial body weight) (n = 6). **(C)** Liver to body weight ratio (n = 6). **(D)** Representative gross anatomy images of livers (n = 6). **(E)** H&E-stained images of livers (scale bar = 100 μm, 100X, n = 6). **(F)** Serum ALT levels (n = 6). **(G)** Serum AST levels (n = 6). **(H)** MT images of livers (scale bar = 100 μm, 100X, n = 6). **(I)** Hepatic Collagen content (n = 6). **(J)** Hepatic Triglyceride content (n = 6). **(K)** Hepatic mRNA expression of pro-inflammatory markers (*Tnfα, Il1β, Ccl2,* and *Ccl5*) and fibrotic markers (*Col1a1, Col3a1, Acta2*, and *Fn1*) shown in heatmap (n = 6). **(L)** Western blots showing hepatic levels of FN, ACTA2, p-ERK, ERK and GAPDH as internal control (n = 6). (M) Liver Cholesterol content (n = 6). **(B)** Data are shown as mean ± SD, one-way ANOVA with Tukey’s correction, statistical significance (*P* values) are shown for comparison between CDHF and CDHF + Fer-1, CDHF + UGP, or CDHF + TGP; **(C,F,G,I,J,M)** Data are shown as box-and-whisker with median (middle line), 25th-75th percentiles (box) and min-max values (whiskers); one-way ANOVA with Tukey’s correction. (ACTA2: Actin alpha cardiac muscle 2; ALT: Alanine transaminase; AST: aspartate aminotransferase; CCL2: C-C motif chemokine ligand 2; CCL5: C-C motif chemokine ligand 5; CDHF: methionine- and choline-deficient diet supplemented with 60 kcal% fat; COL1A1: Collagen type I alpha 1 chain; COL3A1: Collagen type III alpha 1 chain; ERK: Extracellular signal-regulated kinase; FN: Fibronectin; GAPDH: Glyceraldehyde-3-phosphate dehydrogenase; P-ERK: Phosphorylated extracellular signal-regulated kinase; IL-1β: Interleukin 1 beta; TGP: Traditional garlic powder; TNF-α: Tumor necrosis factor alpha; UGP: Ultrafine garlic powder).

We then established 2 mouse models of NASH in order to evaluate the efficacy of UGP as both preventative and therapeutic interventions. Mice fed with CDHF diet induced hepatocyte damage due to excessive fat accumulation, leading to weight loss in the absence of insulin resistance, thus developing acute and severe onset NASH. CDHF mice were intraperitoneally injected with Fer-1 three times per week, while UGP and TGP were administered intragastrically (Figure 5A). CDHF resulted in a significant reduction in the mice body weight (*P* < 0.0001) and a significant increase in the mice liver-to-body weight ratio (*P* < 0.0001) (Figures 5B,C). The observed characteristics were alleviated in mice treated with UGP and Fer-1. The healthy liver undergoes a gradual color transformation from dark brown to orange due to the accumulation of lipids, as indicated by gross anatomy (Figure 5D). Histological analysis of H&E-stained mice liver and elevated serum ALT, AST, γ-GT and ALP levels confirmed steatosis and liver damage in CDHF mice, which were further improved by UGP and Fer-1 treatment (Figures 5E–G; Supplementary Figure S3). Collagen deposition in pericortical and mesenchymal regions was detected by MT staining, and UGP and Fer-1 could significantly reduce CDHF-induced collagen deposition in mice liver (Figure 5H). There was also a significant suppression of markers of fibrosis (Collagen and hepatic triglyceride) in mice treated with UGP and Fer-1 (Figures 5I,J). The upregulation of pro-inflammatory (*Tnfα*, *Il1β*, *Ccl2*, and *Ccl5*) and fibrosis (*Col1a1*, *Col3a1*, *Acta2*, and *Fn1*) genes in CDHF mice was substantially reduced by UGP and Fer-1 (Figure 5K; Supplementary Figure S4). Consistent with the above histological and molecular analyses, ACTA2 and FN proteins were greatly elevated in the liver of CDHF mice, which were further abated by UGP and Fer-1 treatment (Figure 5L). At the signaling level, CDHF diet stimulated the activation of ERK, which was prevented by UGP and Fer-1 (Figure 5L). UGP and Fer-1 have a tendency to reduce liver fat, in other words, lowering cholesterol levels in the liver (Figure 5M). All these indicators suggest that UGP is more preventive than TGP.

### 3.5 UGP protects against ferroptosis in CDHF-induced acute liver injury model

**FIGURE 6:**
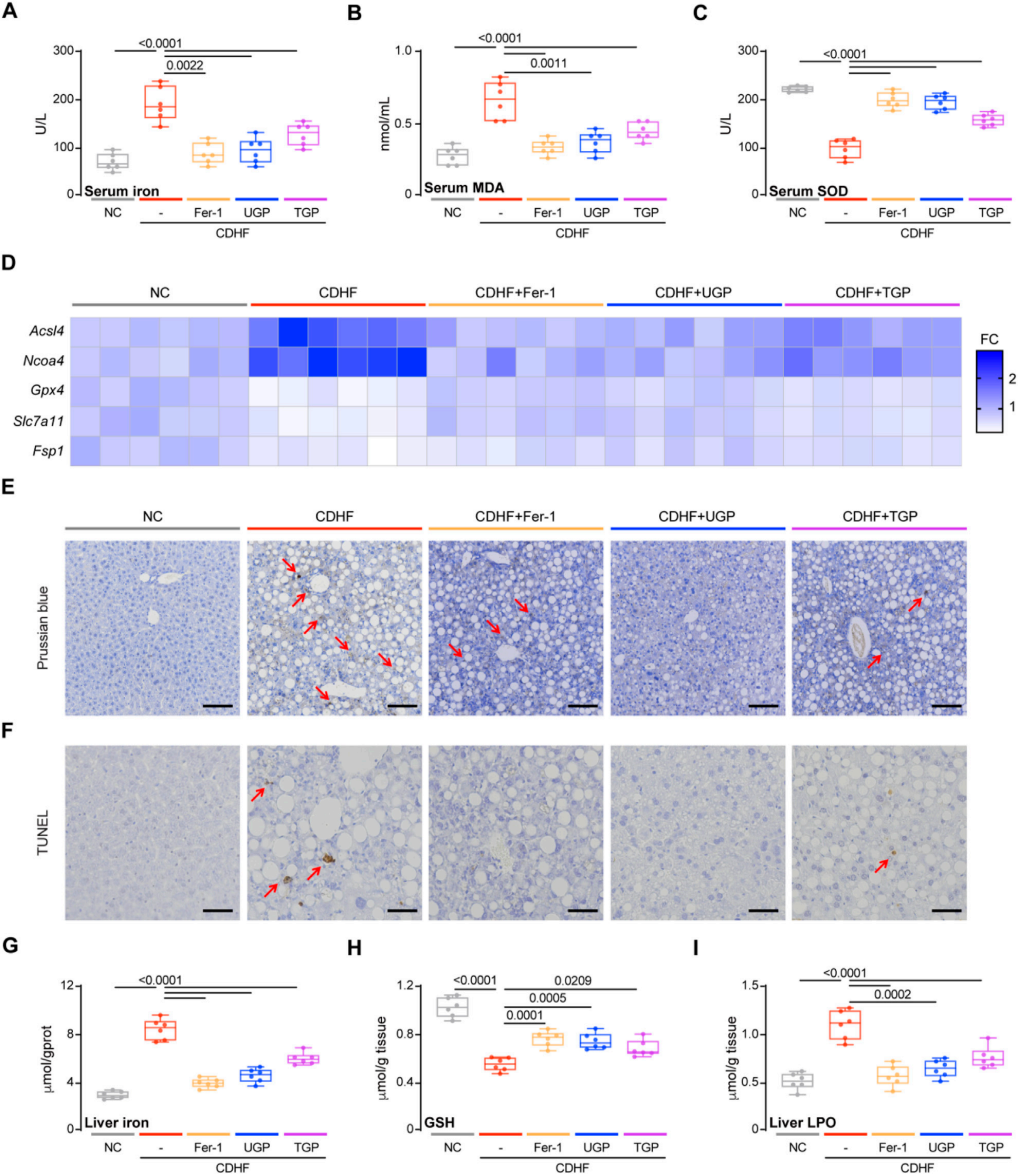
UGP protects against ferroptosis in CDHF-induced acute liver injury model. **(A)** Serum Iron (Fe^3+^+Fe^2+^) levels (n = 6). **(B)** Serum MDA levels (n = 6). **(C)** Serum SOD levels (n = 6). **(D)** Hepatic mRNA expression of ferroptosis markers (*Acsl4, Ncoa4, Gpx4, Slc7a11,* and *Fsp1*) shown in heatmap (n = 6). **(E)** PB-stained images of livers (scale bar = 100 μm, 100X, n = 6). **(F)** TUNEL stained iamges of Livers (scale bar = 50 μm, 200X, n = 6). **(G)** Liver Iron (Fe^3+^+Fe^2+^) levels (n = 6). **(H)** Liver GSH content (n = 6). **(I)** Liver LPO content (n = 6). **(A–C)** and **(G–I)** Data are shown as box-and-whisker with median (middle line), 25th-75th percentiles (box) and min-max values (whiskers); one-way ANOVA with Tukey’s correction. (CDHF: metionine- and choline-deficient diet supplemented with 60 kcal% fat; MDA: malondialdehyde; SOD: Superoxide dismutase; TGP: Traditional garlic powder; UGP:Ultrafine garlic powder).

Our *in vitro* experiments had demonstrated the lipid peroxide and cellular Fe^2+^ accumulation in palmitate-induced hepatocyte lipotoxicity and confirmed the potential role of UGP in ameliorating erastin-induced hepatocyte ferroptosis. To further verify our hypothesis, we examined ferroptosis markers in CDHF-fed mice. NAFLD patients possessed higher levels of circulating iron, and we also observed increased serum iron level in CDHF-induced mice acute liver injury model. MDA and SOD levels reflect the degree of lipid peroxidation in the body, with the former reflecting the severity of free radical attack on human cells while the latter reflecting the body’s ability to scavenge oxygen free radicals. UGP treatment significantly reduced serum iron and MDA levels and restored serum SOD levels compared to TGP (Figures 6A–C). In mice administered UGP and Fer-1, the upregulated genes responsible for ferroptosis (*Acsl4* and *Ncoa4*) were drastically reduced, while the downregulated genes (*Gpx4*, *Slc7a11*, and *Fsp1*) were considerably restored (Figure 6D; Supplementary Figure S5). Ferroptosis status was assessed using PB staining (Figure 6E) while TUNEL staining was conducted to detect cell death (Figure 6F). Hepatic iron deposition was detected by histological PB staining and colorimetric tissue iron analysis, and UGP could greatly prevent the iron accumulation in CDHF-fed mice liver (Figures 6E,G). GSH depletion and GPX4 inactivation are thought to be necessary to promote lipid peroxidation reactions during ferroptosis. UGP treatment restored hepatic GSH level and suppressed LPO levels to a greater extent than TGP, indicating that CDHF-induced lipid peroxidation was greatly improved (Figures 6H,I). Overall, these data suggest a significant improvement of liver ferroptosis by UPG in CDHF mice, in keeping with our finding that UGP inhibited lipid peroxidation and ferroptosis in lipotoxic hepatocytes.

### 3.6 UGP protects against liver inflammation and fibrosis in HFD-induced chronic liver injury model

**FIGURE 7:**
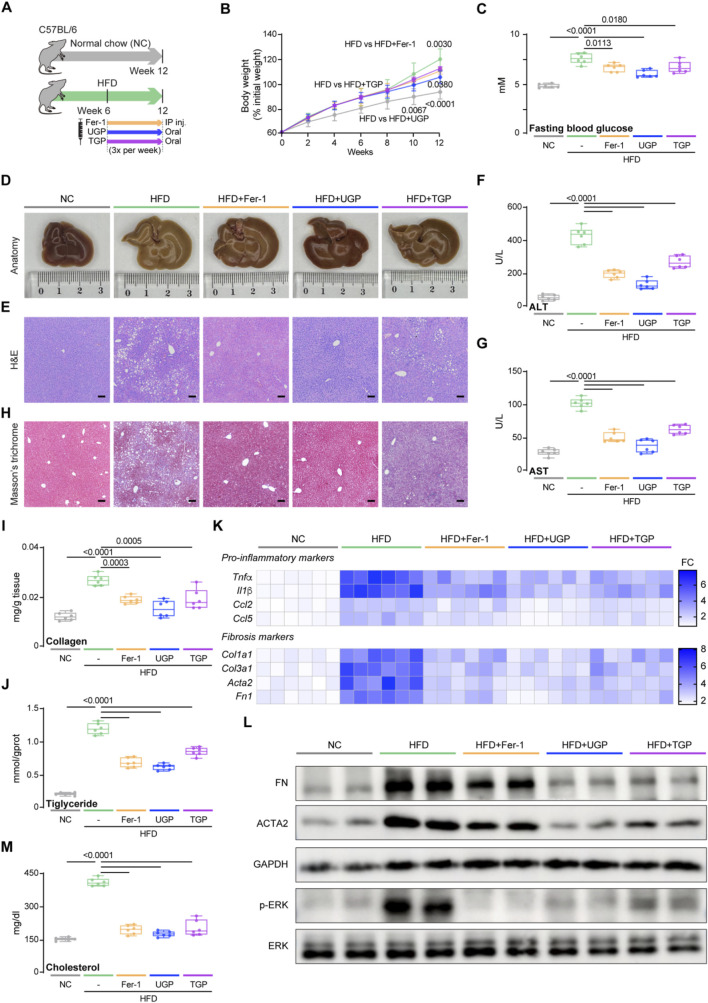
UGP protects against liver inflammation and fibrosis in HFD-induced chronic liver injury model. **(A)** Schematic of HFD feeding in mice treated with Fer-1, UGP and TGP. **(B)** Mice body weight (shown as a percentage (%) of initial body weight) (n = 6). **(C)** Fasting blood glucose level (n = 6). **(D)** Representative gross anatomy images of livers (n = 6). **(E)** H&E-stained images of livers (scale bar = 100 μm, 100X, n = 6). **(F)** Serum ALT levels (n = 6). **(G)** Serum AST levels (n = 6). **(H)** MT images of livers (scale bar = 100 μm, 100X, n = 6). **(I)** Hepatic Collagen content (n = 6). **(J)** Hepatic Triglyceride content (n = 6). **(K)** Hepatic mRNA expression of pro-inflammatory markers (*Tnfα, Il1β, Ccl2,* and *Ccl5*) and fibrotic markers (*Col1a1, Col3a1, Acta2,* and *Fn1*) shown in heatmap (n = 6). **(L)** Western blots showing hepatic levels of FN, ACTA2, p-ERK, ERK and GAPDH as internal control (n = 6). (M) Liver Cholesterol content (n = 6). **(B)** Data are shown as mean ± SD, one-way ANOVA with Tukey’s correction, statistical significance (*P* values) are shown for comparison between HFD and HFD + Fer-1, HFD + UGP, or HFD + TGP; **(C,F,G,I,J,M)** Data are shown as box-and-whisker with median (middle line), 25th-75th percentiles (box) and min-max values (whiskers); one-way ANOVA with Tukey’s correction. (ACTA2: Actin alpha cardiac muscle 2; ALT: Alanine transaminase; AST: aspartate aminotransferase; CCL2: C-C motif chemokine ligand 2; CCL5: C-C motif chemokine ligand 5; COL1A1: Collagen type I alpha 1 chain; COL3A1: Collagen type III alpha 1 chain; ERK: Extracellular signal-regulated kinase; FN: Fibronectin; GAPDH: Glyceraldehyde-3-phosphate dehydrogenase; HFD: diet with 60 kcal% fat; P-ERK: Phosphorylated extracellular signal-regulated kinase; IL-1β: Interleukin 1 beta; TGP: Traditional garlic powder; TNF-α: Tumor necrosis factor alpha; UGP:Ultrafine garlic powder).

In a subsequent experiment, HFD was utilized to induce NASH. The HFD model correlates with obesity, hyperlipidemia, elevated glucose levels, and insulin resistance, replicating typical forms of NASH observed in humans with diabetes. Six weeks after starting the HFD diet, mice were treated with UGP and TGP, and Fer-1 for another 6 weeks (Figure 7A). Mice fed HFD for 12 weeks exhibit obesity and elevated blood glucose levels due to the synthesis of excess fat, which could be greatly improved by UGP and Fer-1 treatment (Figures 7B,C). Through gross morphological, histological, and molecular analyses, mice fed with HFD diet exhibited severe steatosis and fibrosis, which were reversed by the treatment of UGP and Fer-1, and the therapeutic efficacy of UGP was superior to TGP (Figures 7D–M). Mice fed an HFD diet showed significant elevated indications of hepatocyte injury, including rising ALT, AST, γ-GT and ALP levels, and massive tissue vacuolization by H&E staining of liver tissue sections, which were further reversed by treating with UGP and Fer-1 (Figures 7E–G; Supplementary Figure S6). The initial presentation of fibrosis in NASH involves early perisinusoidal fibrosis, marked by balloon-like degeneration of the hepatocytes surrounded by the production of collagen strands. This progresses to progressive perisinusoidal fibrosis and then to bridging fibrosis, ultimately leading to cirrhosis. The ameliorating of fibrosis in UGP and Fer-1 treated mice was confirmed by MT staining and quantitative analysis of hepatic collagen and triglycerides (Figures 7H–J). Additionally, similar to CDHF, HFD also caused dysregulated expression of pro-inflammatory (*Tnfα*, *Il1β*, *Ccl2*, and *Ccl5*) and fibrotic (*Col1a1*, *Col3a1*, *Acta2*, and *Fn1*) genes and proteins, with both molecular phenotypes returning to baseline levels following UGP administration (Figures 7K,L; Supplementary Figure S7). The phosphorylation of ERK signaling induced by HFD was restored by UGP and Fer-1 treatment, indicating consistent signaling alterations (Figure 7L). Moreover, liver cholesterol was also reduced by UGP and Fer-1 (Figure 7M). HFD mice treated with UGP exhibited superior capability in abating liver damage and fibrosis when comparing to TGP. Taken together, these data suggest that UGP improved liver metabolism and reversed the established inflammation and fibrosis in NASH mice.

### 3.7 Transcriptomics analysis of mice liver for the role of UGP in HFD-induced chronic liver injury

**FIGURE 8:**
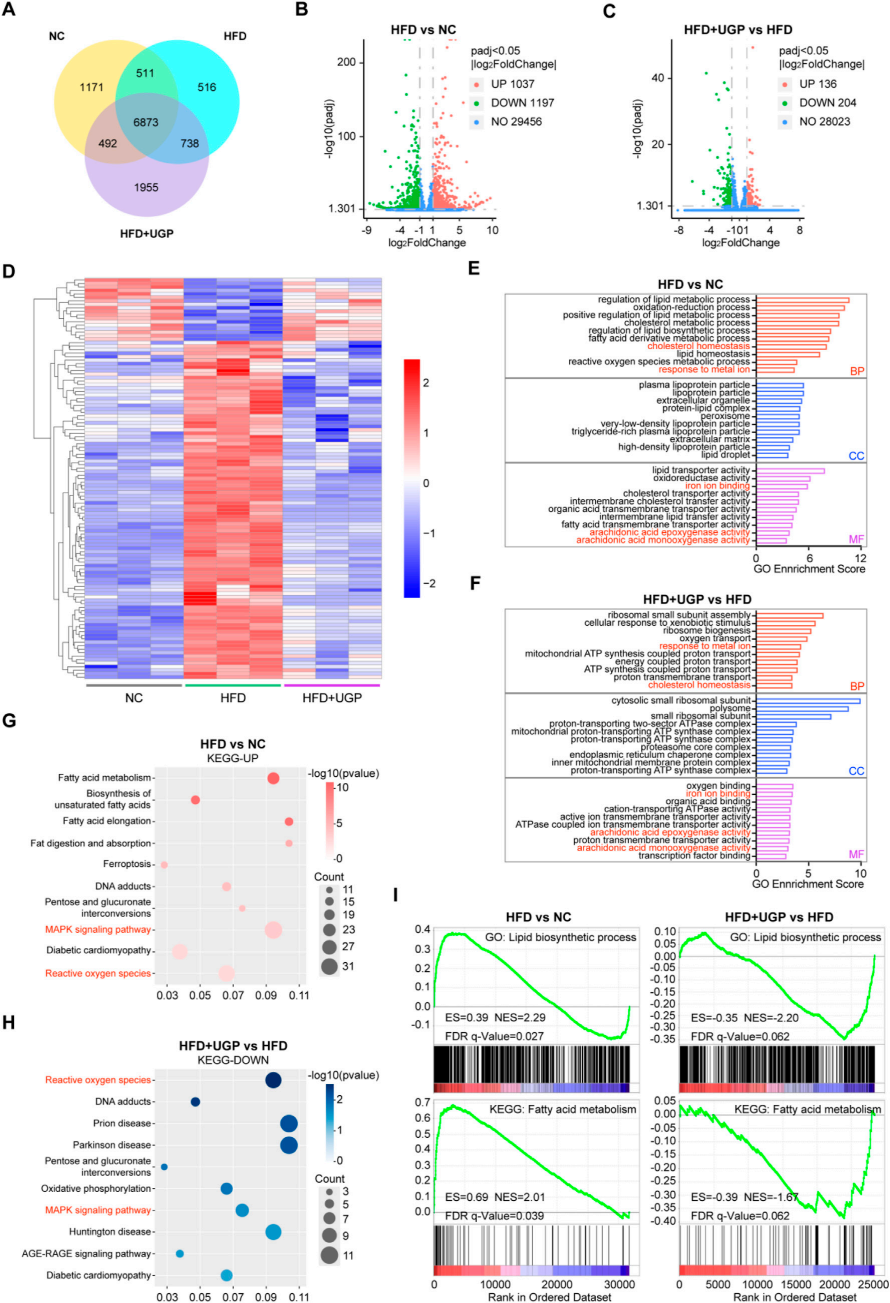
Transcriptomics analysis of mice liver for the role of UGP in HFD-induced chronic liver injury. **(A)** Venn diagram illustrating the number of genes co-expressed in NC, HFD, and HFD + UGP groups. The volcano map displaying DEGs between NC and HFD **(B)**, or HFD and HFD + UGP **(C)** groups. **(D)** The heatmap showing DEGs among NC, HFD and HFD + UGP groups. **(E)** GO enrichment analysis of DEGs between NC and HFD groups. **(F)** GO enrichment analysis of DEGs between HFD and HFD + UGP groups. **(G)** KEGG enrichment analysis of upregulated pathways in HFD compared to NC in accordance with DEGs. **(H)** KEGG enrichment analysis of downregulated pathways in HFD + UGP compared to HFD in accordance with DEGs. **(I)** GSEA enrichment analysis identified the potential GO or KEEG pathways. (CDHF: methionine- and choline-deficient diet supplemented with 60 kcal% fat; HFD: diet with 60 kcal% fat; TGP: Traditional garlic powder; UGP:Ultrafine garlic powder).

To systematically elucidate the underlying mechanism of the beneficial role of UGP in NASH, transcriptome RNA-seq was performed on the livers of NC, HFD, and UGP-treated HFD mice. First, a quantitative analysis was performed on each group’s gene expression. A Venn map was used to depict the number of genes that were uniquely expressed in each group. This map revealed 6873 genes that crossovered between NC, HFD, and HFD + UGP groups (Figure 8A). Next, the entire set of DEGs between NC, HFD, and HFD + UGP groups was displayed on the volcano plot. When compared to NC, 1037 genes were upregulated and 1197 genes were downregulated in HFD group (Figure 8B). While as compared to HFD, 136 genes were upregulated and 204 were downregulated in HFD + UGP group (Figure 8C). The heatmap displayed the genetic similarities between the NC and HFD + UGP groups, which are different from HFD group (Figure 8D). GO analysis indicated that UGP treatment in HFD mice led to specific enrichment of DEGs in biological process including response to metal ion and cholesterol homeostasis, and molecular functions including iron ion binding, arachidonic acid epoxygenase activity and arachidonic acid monooxygenase activity, as compared with NC controls (Figures 8E,F). Subsequent KEGG enrichment analysis revealed that fatty acid metabolism, ferroptosis, pentose and glucuronate interconversions, MAPK signaling pathway and ROS were upregulated in HFD group (Figure 8G). On the other hand, UGP treatment significantly suppressed the activation of MAPK signaling pathway and ROS production, in line with the aforesaid molecular analysis (Figure 8H). To further explain the above results, we proofread the GO and KEGG enrichment analyses by GSEA tool. And the therapeutic role of UGP in HFD-induced mice with NASH was evidenced by regulating lipid biosynthesis process and fatty acid metabolism (Figure 8I).

### 3.8 UGP protects against ferroptosis in HFD-induced chronic liver injury model

**FIGURE 9:**
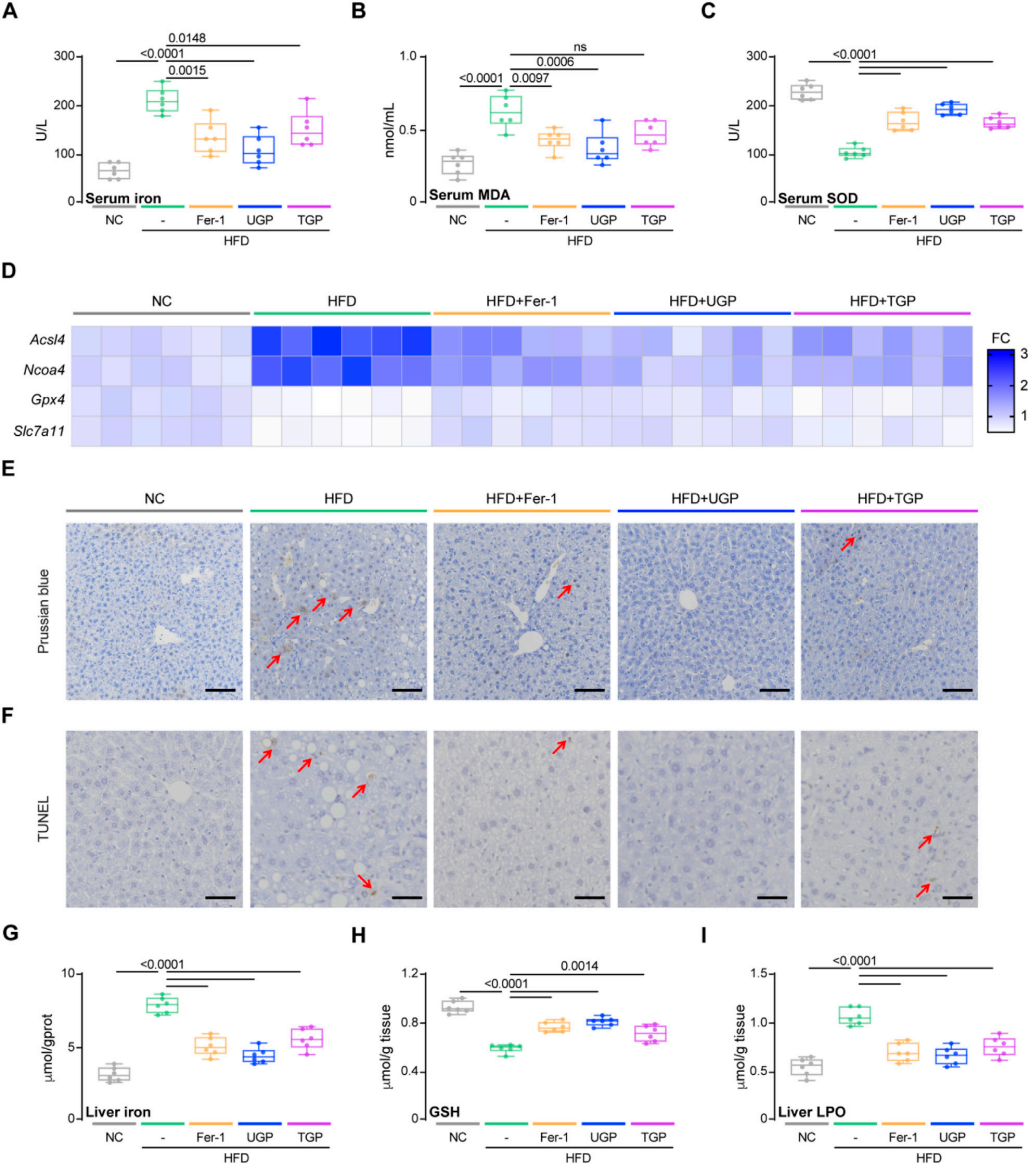
UGP protects against ferroptosis in HFD-induced chronic liver injury model. **(A)** Serum Iron (Fe^3+^+Fe^2+^) levels (n = 6). **(B)** Serum MDA levels (n = 6). **(C)** Serum SOD levels (n = 6). **(D)** Hepatic mRNA expression of ferroptosis markers (*Acsl4, Ncoa4, Gpx4,* and *Slc7a11*) shown in heatmap (n = 6). **(E)** PB-stained images of livers (scale bar = 100 μm, 100X, n = 6). **(F)** TUNEL-stained iamges of Livers (scale bar = 50 μm, 200X, n = 6). **(G)** Liver Iron (Fe^3+^+Fe^2+^) levels (n = 6). **(H)** Liver GSH content (n = 6). **(I)** Liver LPO content (n = 6). **(A–C)** and **(G–I)** Data are shown as box-and-whisker with median (middle line), 25th-75th percentiles (box) and min-max values (whiskers); one-way ANOVA with Tukey’s correction. (HFD: diet with 60 kcal% fat; MDA: malondialdehyde; SOD: Superoxide dismutase; TGP: Traditional garlic powder; UGP:Ultrafine garlic powder).

Based on the previous *in vivo* and *in vitro* experiments, it was concluded that UGP can improve lipotoxic hepatocyte injury by inhibiting ferroptosis. In HFD-induced chronic liver injury model, we also measured the ferroptosis parameters. In comparison to NC, HFD diet can induce ferroptosis in mice, as can be detected by molecular and histological assays. UGP could greatly decrease the extent of ferroptosis in mice, comparable to Fer-1 (Figure 9; Supplementary Figure S8). In mice fed with HFD diet, circulating levels of iron and MDA were significantly increased, accompanied by a notable decrease in SOD, and all explained the ROS production in NASH mice (Figures 9A–C). As expected, UGP and Fer-1 restored the increasing (*Acsl4* and *Ncoa4*) and decreasing (*Gpx4* and *Slc7a11*) of genes associated with ferroptosis (Figure 9D; Supplementary Figure S8). Besides, TUNEL staining showed the occurrence of apoptosis in the livers of HFD mice, PB staining of liver sections and quantitative analysis of iron content in liver tissues also reflected the large amount of iron deposition in the liver of HFD mice (Figures 9E–G). All the changes in these parameters gradually returned to normal under the intervention of Fer-1 and UGP, and TGP was not as effective as UGP treatment (Figures 9A–G). Furthermore, the accumulation of excess fat in hepatocytes triggered oxidative stress and generated a significant amount of ROS, impeding the cellular redox process and the decrease in GSH levels, which were normalized by UGP and Fer-1 (Figure 9H). Liver LPO levels were also reduced by UGP, indicating the amelioration of lipid peroxidation (Figure 9I). These data overall demonstrated that ferroptosis plays an important role in the development of NASH, and UGP can prevent or even reverse the development and progression of NASH by inhibiting hepatocyte ferroptosis-induced liver steatosis, inflammation and fibrosis.

## 4 Discussion

Iron overload is a prominent characteristic of ferroptosis, which promotes the accumulation of lipid ROS by generating hydroxyl and alkoxyl radicals *via* the Fenton reaction, hence increasing oxidative damage (Dixon et al., 2012). Ferroptotic cell death can be blocked by regulating genes related to iron overload or using iron-chelating agents, indicating a central role of iron in the regulation and execution of ferroptosis (Tang et al., 2021). Numerous studies have demonstrated the significance of iron overload and ferroptosis in various liver disease models, such as hemochromatosis, alcohol-related liver disease (ALD), hepatitis C virus (HCV) infection, NASH (Qi et al., 2020), and hepatocellular carcinoma (HCC). Ferroptosis has recently been identified as a potential therapeutic target for patients with NASH (Shu et al., 2022; Nelson et al., 2011; Wei et al., 2020), and inhibiting hepatic iron accumulation has demonstrated notable effectiveness in ameliorating insulin resistance, promoting insulin secretion, and recovering abnormal liver enzyme levels (Mou et al., 2019). Evidence has demonstrated that ferroptosis serves as a trigger for the initiation of inflammation in steatohepatosis, and therefore inhibiting ferroptosis is a goal for the prevention and treatment of NASH. Qi et al. (Qi et al., 2020) discovered that DFO, an iron chelating agent, was able to alleviate the progression of NASH in mice by inhibiting GPX4 activation. They also confirmed that the regulation of ferroptosis had an impact on palmitic acid-induced hepatocyte death.

Our data show a central importance of ferroptosis in hepatocytes for multiple NASH pathologies. This effect was confirmed by establishing a palmitic acid-induced hepatic lipotoxicity model and two types of high fat diet-induced mouse NASH models. Fer-1 significantly improved lipotoxic hepatocyte injury induced by palmitic acid in THLE2, as evidenced by increased cell viability and decreased levels of ROS and lipid peroxidation. Meanwhile, Fer-1 treatment significantly reduced hepatic iron overload, inflammation, fibrosis, and ferroptosis in CDHF and HFD diet-fed mice. Fer-1 has been reported to improve inflammation, fibrosis, and hepatic injury in NASH mice fed with MCD diet (Li XY. et al., 2020). Mohs et al. proved that Fer-1 was able to increase GPX4 levels, reduce lipid peroxidation, and improve antioxidant capacity, particularly by increasing the levels of CAT, NQO1, and HO1. NQO1 and HO1 are downstream of the Nrf2 antioxidant genes, regulating lipid metabolism and protecting mice from lipotoxicity. They also control fibrogenesis and carcinogenesis during steatohepatitis (Mohs et al., 2021). Additionally, Fer-1 treatment could activate peptide tyrosine tyrosine (PYY) and elevate *Pparg* mRNA expression, regulating glucose and lipid homeostasis (Janani and Ranjitha Kumari, 2015).

As one of the oldest herbs, the medicinal value of garlic has been widely recognized in several civilizations (Rivlin, 2001). Because of its ability to move qi and blood, warm the spleen and stomach, eliminate stagnation and dissipate stagnation, detoxify and kill worms, as well as being used to repel worms and anti-infection (Agarwal, 1996). Garlic has been shown to have therapeutic effects on metabolic disorders such as dyslipidemia and diabetes, exhibiting significant antioxidant activity. In a recent clinical experiment conducted by Soleimani et al., it was demonstrated that the use of garlic powder led to a notable enhancement in hepatic steatosis and the associated comorbidities in NAFLD patients (Soleimani et al., 2021). In addition, Kim et al. (Kim et al., 2017) reported a positive effect on ALT levels in individuals with moderate liver dysfunction who received fermented garlic extract in a double-blind randomized controlled experiment. S-allymercaptocysteine (SAMC), the main bioactive compound found in garlic, is a precursor compound of SAC responsible for its therapeutic effects, has the ability to reduce fibrogenic factors such as transforming growth factor-β1 (TGF-β1), α-smooth muscle actin (α-SMA), and inflammatory cytokines that activate HSCs, resulting in the accumulation of collagen (Xiao et al., 2013b). Additionally, SAMC can reduce inflammatory responses by targeting NF-κB, and improve lipid homeostasis and insulin resistance through regulating AMPK and IRS-1/PI3K/Akt pathways (Xiao et al., 2013a). Garlic essential oil (GEO) and its principal active compound (diallyl disulfide, DADS) exhibited a hepatoprotective effect in HFD-fed obese mice with NAFLD. The impact was dependent on the dosage of the substances. GEO and DADS decreased the weight of adipose tissue generated by HFD and ameliorated insulin abnormalities by reducing blood glucose levels (Lai et al., 2014). As one of the major compounds in garlic, Alliin has been shown to be effective in preventing triglyceride accumulation and hepatic steatosis in HepG2 cells and C57BL/6N mice induced by endoplasmic reticulum stress (Yun and Lee, 2022). Hwang et al. demonstrated that SAC can prevent lipid accumulation and lipotoxicity caused by free fatty acids (FFA), reactive oxygen species (ROS) production, and subsequent cell death of hepatocytes (Hwang et al., 2013). In this study, *in vitro* experiments showed that garlic powder effectively prevented palmitic acid- or erastin-induced hepatocyte lipotoxicity and ferroptosis, through reducing ROS and lipid peroxidation, and repairing mitochondrial damage. By establishing *in vivo* NASH models, we have confirmed that garlic powder could improve hepatic steatosis, inflammation, and fibrosis in mice that were fed with CDHF and HFD diets.

Modern advanced technology has the capability to transform traditional Chinese medicines into powders of extremely small size, known as micron-sized ultrafine powders. Ultrafine powder has notable attributes such as extensive surface area, heightened surface reactivity, rapid chemical kinetics, and distinctive thermal, mechanical, optical, and magnetic properties. This formulation also exhibits high water solubility, little loss of functional components, and a substantial level of utilization (Li Y. et al., 2020; Muttakin et al., 2015; Wang et al., 2023). Consequently, it has the potential to enhance the efficacy of medications at the cellular and molecular levels. Previous study discovered that ultrafine Dendrobium officinale powder was more efficient than the traditional Dendrobium officinale decoction in maintaining the equilibrium of intestinal microbiota and intestinal enzyme activity in mice with spleen deficiency and constipation (Cao et al., 2014). In this study, we used UGP produced by a high-speed and energy particle collision equipment, which was distinct from TGP. Smaller particle size was confirmed by particle size analyzer and SEM images. In addition, ultrafine powder technology also improved solubility and dispersibility in distinct solvents with hydroxyl functional groups and excellent hydrophilic properties, and enhanced the *ex vivo* antioxidant capability of garlic. Although this study did not directly quantify cellular uptake or intracellular exposure of UGP and TGP, multiple lines of indirect evidence—including markedly reduced particle size, surface hydroxylation, decreased contact angle, improved solubility and dispersibility, and GC-MS/LC-MS confirmed enrichment of key organosulfur constituents-collectively support the inference that UGP exhibits greater bioavailability than TGP. Our *in vitro* experiments have confirmed that UGP was more effective than TGP in inhibiting lipid peroxidation, ROS production, lipotoxicity, and ferroptosis in THLE2 cells and subsequent activation of HSCs. In addition, UGP also significantly reduced liver damage and collagen deposition in CDHF- and HFD-fed NASH mice, as compared with TGP.

To further investigate the underlying mechanism of UGP against HFD-induced mouse NASH model, we conducted RNA-seq analysis. It has been suggested that the disruption of metal detoxification processes in the liver may be associated with the development of NAFLD through oxidative stress (Feldman et al., 2015). Excess iron in the body increases oxidative stress in the endoplasmic reticulum, triggering liver damage through the formation of hydroxyl radicals *via* the Fenton reaction. This is in line with our GO enrichment analysis, which showed significant changes in the response to metal ion and the binding of iron ion between NC and HFD groups. Our findings indicate that UGP potentially exerts its effects via the MAPK signaling pathway and ROS production. The MAPK signaling pathway is primarily involved in regulating inflammation and fatty acid metabolism (Kyriakis and Avruch, 2012). Research has demonstrated that suppressing the MAPK signaling pathway in NASH rats can alleviate liver fibrosis and hepatocyte apoptosis (Shen et al., 2019). Further analysis of Western blot images revealed that UGP effectively hindered the phosphorylation of ERK, thereby reducing the progression of NASH. Our previous study has shown the key role of ERK signaling in the pathophysiology of NASH (Dong et al., 2021). Other signaling pathways, including JNK/p38 MAPK, PI3K–AKT, NF-κB, and TGF-β/Smad, also contribute to the pathogenesis of NASH. These pathways merit systematic investigation in future studies. Potential approaches include time-resolved phosphorylation profiling, pathway-specific perturbation experiments, and tissue-resolved analyses to delineate their dynamic and spatial regulatory roles. TGF-β1 and its downstream SMAD and ERK signaling pathways are markedly activated when liver damage progresses to HCC. Prolonged ERK phosphorylation is frequently associated with oxidative damage and inflammation (Yan et al., 2017). Moreover, excessive ROS and dysregulated cholesterol homeostasis can initiate a persistent and uncontrolled inflammatory response, which is accompanied by the release of pro-inflammatory cytokines from immune cells. According to our model, UGP has the potential to improve high blood sugar levels and lipid metabolism in mice by blocking the MAPK/ERK pathway, which in turn reduces liver damage.

This study did not directly confirm a physical interaction between individual UGP constituents and ERK. To address this limitation, a complementary strategy involving targeted identification and functional validation is recommended. First, candidate compounds identified by GC-MS or LC-MS can be prioritized through *in silico* approaches, including molecular docking and molecular dynamics simulations. Direct binding can then be assessed using cellular thermal shift assays, differential scanning fluorimetry, or biophysical techniques such as surface plasmon resonance and isothermal titration calorimetry, followed by functional validation using *in vitro* kinase assays with purified ERK. Where appropriate, affinity-based pull-down proteomics may be employed to identify additional binding partners. Indirect mechanisms should also be considered, such as modulation of ERK activity via upstream kinases or phosphatases, or through changes in the cellular redox state. Together, these studies would help differentiate direct ligand–kinase interactions from indirect regulatory effects, providing more definitive molecular evidence for the ERK-related phenotypes observed in this work.

## 5 Conclusion

In conclusion, our study suggests that UGP reduces ROS production by inhibiting ferroptosis, which is achieved by restoring GSH depletion and reducing Fe^2+^ production, thereby inhibiting the activation of MAPK/ERK pathway, regulating lipid metabolism, and preventing excessive lipid accumulation, which in turn reduces HSC activation and immune cell recruitment, suppressing the development and progression of inflammation and fibrosis, and ultimately ameliorating NASH. This research demonstrates the promising therapeutic role of UGP in NASH and gives insights and research directions for the future development of new strategies for prevention and treatment of NASH with natural herbs from the perspective of ultrafine powder technology.

## Data Availability

The data is deposited in CNCBdb repository, accession number CNP0008355. Available at: https://db.cngb.org/data_resources/?query=CNP0008355.

## Glossary

a- SMA: a-smooth muscle actin
ALD: alcohol-related liver disease
ALT: alanine transaminase
AST: aspartate aminotransferase
ALP: alkaline phosphatase
DADS: diallyl disulfide
FFA: free fatty acids
FN: fibronectin
Fer-1: ferrostatin-1
γ-GT: γ-Glutamyltransferase
GSH: glutathione
GEO: garlic essential oil
GSAC: g-glutamyl-S-allyl-cysteine
HSCs: hepatic stellate cells
H&E: hematoxylin and eosin
HCV: hepatitis C virus
HCC: hepatocellular carcinoma
LPO: lipid peroxides
MDA: malondialdehyde
MT: masson’s trichrome
NAFLD: non-alcoholic fatty liver disease
NASH: non-alcoholic steatohepatitis
PFA: paraformaldehyde
PB: prussian blue
ROS: reactive oxygen species
SOD: superoxide dismutase
SAC: S-Allyl cysteine
SAMC: S-allymercaptocysteine
TGP: traditional garlic powder
TEM: transmission electron microscopy
TGF-β1: transforming growth factor-β1
UGP: ultrafine garlic powder.

## References

[B1] Abdel Halim A. S. Ali M. A. M. Inam F. Alhalwan A. M. Daoush W. M. (2024). Fe(3)O(4)-Coated CNTs-Gum Arabic nano-hybrid composites exhibit enhanced anti-leukemia potency against AML cells *via* ROS-mediated signaling. Int. J. Nanomedicine 19, 7323–7352. 10.2147/IJN.S467733 39055376 PMC11269411

[B2] Agarwal K. C. (1996). Therapeutic actions of garlic constituents. Med. Res. Rev. 16 (1), 111–124. 10.1002/(SICI)1098-1128(199601)16:1<111::AID-MED4>3.0.CO;2-5 8788216

[B3] Ankri S. Mirelman D. (1999). Antimicrobial properties of allicin from garlic. Microbes Infect. 1 (2), 125–129. 10.1016/s1286-4579(99)80003-3 10594976

[B4] Cai D. S. Yuan M. S. Frantz D. F. Melendez P. A. Hansen L. Lee J. (2005). Local and systemic insulin resistance resulting from hepatic activation of IKK-β and NF-κB. Nat. Med. 11 (2), 183–190. 10.1038/nm1166 15685173 PMC1440292

[B5] Cao R. Wang H. Wu W. Tan Z. Xiao X. Wu L. (2014). Effect of ultra-micro Dendrobium officinale powder on the intestinal microbiota and enzyme activities in mice with spleen -deficiency constipation. Chin. J. Microecol. 26 (9), 1011–1015.

[B6] Chalasani N. Younossi Z. Lavine J. E. Diehl A. M. Brunt E. M. Cusi K. (2012). The diagnosis and management of non-alcoholic fatty liver disease: practice guideline by the American gastroenterological association, American association for the study of liver diseases, and American college of gastroenterology. Gastroenterology 142 (7), 1592–1609. 10.1053/j.gastro.2012.04.001 22656328

[B7] Choi J. Choi H. Chung J. Y. (2023). Icariin supplementation suppresses the markers of ferroptosis and attenuates the progression of nonalcoholic steatohepatitis in mice fed a methionine choline-deficient diet. Int. J. Mol. Sci. 24 (15), 12510. 10.3390/ijms241512510 37569885 PMC10419585

[B8] Dixon S. J. Lemberg K. M. Lamprecht M. R. Skouta R. Zaitsev E. M. Gleason C. E. (2012). Ferroptosis: an iron-dependent form of nonapoptotic cell death. Cell 149 (5), 1060–1072. 10.1016/j.cell.2012.03.042 22632970 PMC3367386

[B9] Dong J. Viswanathan S. Adami E. Singh B. K. Chothani S. P. Ng B. (2021). Hepatocyte-specific IL11 cis-signaling drives lipotoxicity and underlies the transition from NAFLD to NASH. Nat. Commun. 12 (1), 66. 10.1038/s41467-020-20303-z 33397952 PMC7782504

[B10] Dong J. Li P. Ji X. Kang Y. Yuan X. Tang J. (2023). Electrons of d-orbital (mn) and p-orbital (N) enhance the photocatalytic degradation of antibiotics by biochar while maintaining biocompatibility: a combined chemical and biological analysis. J. Hazard Mater 451, 131083. 10.1016/j.jhazmat.2023.131083 36878031

[B11] Ernst E. (1997). Can allium vegetables prevent cancer? Phytomedicine 4 (1), 79–83. 10.1016/S0944-7113(97)80032-3 23195250

[B12] Feldman A. Aigner E. Weghuber D. Paulmichl K. (2015). The potential role of iron and copper in pediatric obesity and nonalcoholic fatty liver disease. Int. Br. 2015, 287401. 10.1155/2015/287401 26273604 PMC4529901

[B13] Friedman S. L. Neuschwander-Tetri B. A. Rinella M. Sanyal A. J. (2018). Mechanisms of NAFLD development and therapeutic strategies. Nat. Med. 24 (7), 908–922. 10.1038/s41591-018-0104-9 29967350 PMC6553468

[B14] Hwang Y. P. Kim H. G. Choi J. H. Do M. T. Chung Y. C. Jeong T. C. (2013). S-Allyl cysteine attenuates free fatty acid-induced lipogenesis in human HepG2 cells through activation of the AMP-Activated protein kinase-dependent pathway. J. Nutr. Biochem. 24 (8), 1469–1478. 10.1016/j.jnutbio.2012.12.006 23465592

[B15] Janani C. Ranjitha Kumari B. D. (2015). PPAR gamma gene--a review. Diabetes Metab. syndr. 9 (1), 46–50. 10.1016/j.dsx.2014.09.015 25450819

[B16] Jiang X. J. Stockwell B. R. Conrad M. (2021). Ferroptosis: mechanisms, biology and role in disease. Nat. Rev. Mol. Cell Biol. 22 (4), 266–282. 10.1038/s41580-020-00324-8 33495651 PMC8142022

[B17] Jiang H. Zhang X. Yang W. Li M. Wang G. Luo Q. (2022). Ferrostatin-1 ameliorates liver dysfunction *via* reducing iron in thioacetamide-induced acute liver injury in mice. Front. Pharmacol. 13, 869794. 10.3389/fphar.2022.869794 35496274 PMC9039014

[B18] Karagodin V. P. Sobenin I. A. Orekhov A. N. (2016). Antiatherosclerotic and cardioprotective effects of time-released garlic powder pills. Curr. Pharm. Des. 22 (2), 196–213. 10.2174/1381612822666151112153351 26561055

[B19] Kechagias S. Ernersson Å. Dahlqvist O. Lundberg P. Lindstroem T. Nystrom F. H. (2008). Fast-food-based hyper-alimentation can induce rapid and profound elevation of serum alanine aminotransferase in healthy subjects. Gut 57 (5), 649–654. 10.1136/gut.2007.131797 18276725 PMC2565580

[B20] Kim H. N. Kang S. G. Roh Y. K. Choi M. K. Song S. W. (2017). Efficacy and safety of fermented garlic extract on hepatic function in adults with elevated serum gamma-glutamyl transpeptidase levels: a double-blind, randomized, placebo-controlled trial. Eur. J. Nutr. 56 (5), 1993–2002. 10.1007/s00394-016-1318-6 27743130

[B21] Kleinfeld A. M. Prothro D. Brown D. L. Davis R. C. Richieri G. V. DeMaria A. (1996). Increases in serum unbound free fatty acid levels following coronary angioplasty. Am. J. Cardiol. 78 (12), 1350–1354. 10.1016/s0002-9149(96)00651-0 8970405

[B22] Kowdley K. V. Belt P. Wilson L. A. Yeh M. M. Neuschwander-Tetri B. A. Chalasani N. (2012). Serum ferritin is an independent predictor of histologic severity and advanced fibrosis in patients with nonalcoholic fatty liver disease. Hepatology 55 (1), 77–85. 10.1002/hep.24706 21953442 PMC3245347

[B23] Kyriakis J. M. Avruch J. (2012). Mammalian mapk signal transduction pathways activated by stress and inflammation: a 10-Year update. Physiol. Rev. 92 (2), 689–737. 10.1152/physrev.00028.2011 22535895

[B24] Lai Y. S. Chen W. C. Ho C. T. Lu K. H. Lin S. H. Tseng H. C. (2014). Garlic essential oil protects against obesity-triggered nonalcoholic fatty liver disease through modulation of lipid metabolism and oxidative stress. J. Agr Food Chem. 62 (25), 5897–5906. 10.1021/jf500803c 24857364

[B25] Lei S. S. Li B. Chen Y. H. He X. L. S. Wang Y. Z. Yu H. H. (2019). Dendrobii officinalis, a traditional Chinese edible and officinal plant, accelerates liver recovery by regulating the gut-liver axis in NAFLD mice. J. Funct. Foods 61, 103458. 10.1016/j.jff.2019.103458

[B26] Li X. Y. Wang T. X. Huang X. M. Li Y. Sun T. G. Zang S. F. (2020a). Targeting ferroptosis alleviates methionine-choline deficient (MCD)-Diet induced NASH by suppressing liver lipotoxicity. Liver Int. 40 (6), 1378–1394. 10.1111/liv.14428 32145145

[B27] Li Y. Li J. Liu B. Lu T. (2020b). Application overview on traditional Chinese medicine with ultra-fine powder. China J. Traditional Chin. Med. Pharm. 35 (9), 4568–4570.

[B28] Li D. Xue X. Li J. Li H. Zhu Q. (2022). Improvement on fluidization and reduction of ultrafine CuO powders with the assistance of iron microspheres. Powder Technol. 411, 117936. 10.1016/j.powtec.2022.117936

[B29] Loomba R. Schork N. Chen C. H. Bettencourt R. Bhatt A. Ang B. (2015). Heritability of hepatic fibrosis and steatosis based on a prospective twin study. Gastroenterology 149 (7), 1784–1793. 10.1053/j.gastro.2015.08.011 26299412 PMC4663110

[B30] Lyu H. Zhang H. Dong J. Shen B. Cheng Z. Yu J. (2024). Pyrolysis temperature matters: biochar-Derived dissolved organic matter modulates aging behavior and biotoxicity of microplastics. Water Res. 250, 121064. 10.1016/j.watres.2023.121064 38154336

[B31] Marjot T. Moolla A. Cobbold J. F. Hodson L. Tomlinson J. W. (2020). Nonalcoholic fatty liver disease in adults: current concepts in etiology, outcomes, and management. Endocr. Rev. 41 (1), 66–117. 10.1210/endrev/bnz009 31629366

[B32] Mohs A. Otto T. Schneider K. M. Peltzer M. Boekschoten M. Holland C. H. (2021). Hepatocyte-specific NRF2 activation controls fibrogenesis and carcinogenesis in steatohepatitis. J. Hepatol. 74 (3), 638–648. 10.1016/j.jhep.2020.09.037 33342543

[B33] Moore M. P. Cunningham R. P. Dashek R. J. Mucinski J. M. Rector R. S. (2020). A fad too far? Dietary strategies for the prevention and treatment of NAFLD. Obes. (Silver Spring) 28 (10), 1843–1852. 10.1002/oby.22964 32893456 PMC7511422

[B34] Mou Y. H. Wang J. Wu J. C. He D. Zhang C. F. Duan C. J. (2019). Ferroptosis, a new form of cell death: opportunities and challenges in cancer. J. Hematol. Oncol. 12 (1), 34. 10.1186/s13045-019-0720-y 30925886 PMC6441206

[B35] Muttakin S. Kim M. S. Lee D. U. (2015). Tailoring physicochemical and sensorial properties of defatted soybean flour using jet-milling technology. Food Chem. 187, 106–111. 10.1016/j.foodchem.2015.04.104 25977004

[B36] Nelson J. E. Wilson L. Brunt E. M. Yeh M. M. Kleiner D. E. Unalp-Arida A. (2011). Relationship between the pattern of hepatic iron deposition and histological severity in nonalcoholic fatty liver disease. Hepatology 53 (2), 448–457. 10.1002/hep.24038 21274866 PMC3058264

[B37] Ng B. Dong J. D'Agostino G. Viswanathan S. Widjaja A. A. Lim W. W. (2019). Interleukin-11 is a therapeutic target in idiopathic pulmonary fibrosis. Sci. Transl. Med. 11 (511), eaaw1237. 10.1126/scitranslmed.aaw1237 31554736

[B38] Pertea M. Pertea G. M. Antonescu C. M. Chang T. C. Mendell J. T. Salzberg S. L. (2015). StringTie enables improved reconstruction of a transcriptome from RNA-Seq reads. Nat. Biotechnol. 33 (3), 290–295. 10.1038/nbt.3122 25690850 PMC4643835

[B39] Qi J. Kim J. W. Zhou Z. X. Lim C. W. Kim B. (2020). Ferroptosis affects the progression of nonalcoholic steatohepatitis *via* the modulation of lipid peroxidation-mediated cell death in mice. Am. J. Pathol. 190 (1), 68–81. 10.1016/j.ajpath.2019.09.011 31610178

[B40] Riazi K. Azhari H. Charette J. H. Underwood F. E. King J. A. Afshar E. E. (2022). The prevalence and incidence of NAFLD worldwide: a systematic review and meta-analysis. Lancet Gastroenterol. 7 (9), 851–861. 10.1016/S2468-1253(22)00165-0 35798021

[B41] Rich N. E. Oji S. Mufti A. R. Browning J. D. Parikh N. D. Odewole M. (2018). Racial and ethnic disparities in nonalcoholic fatty liver disease prevalence, severity, and outcomes in the United States: a systematic review and meta-analysis. Clin. Gastroenterol. H. 16 (2), 198–210.e2. 10.1016/j.cgh.2017.09.041 28970148 PMC5794571

[B42] Rivlin R. S. (2001). Historical perspective on the use of garlic. J. Nutr. 131 (3), 951S–954S. 10.1093/jn/131.3.951S 11238795

[B43] Rouf R. Uddin S. J. Sarker D. K. Islam M. T. Ali E. S. Shilpi J. A. (2020). Antiviral potential of garlic (Allium sativum) and its organosulfur compounds: a systematic update of pre-clinical and clinical data. Trends Food Sci. Technol. 104, 219–234. 10.1016/j.tifs.2020.08.006 32836826 PMC7434784

[B44] Sangouni A. A. Mohammad Hosseini Azar M. R. Alizadeh M. (2020). Effect of garlic powder supplementation on hepatic steatosis, liver enzymes and lipid profile in patients with non-alcoholic fatty liver disease: a double-blind randomised controlled clinical trial. Br. J. Nutr. 124 (4), 450–456. 10.1017/S0007114520001403 32312333

[B45] Shang A. Cao S. Y. Xu X. Y. Gan R. Y. Tang G. Y. Corke H. (2019). Bioactive compounds and biological functions of garlic (Allium sativum L.). Foods 8 (7), 246. 10.3390/foods8070246 31284512 PMC6678835

[B46] Shen X. T. Guo H. Y. Xu J. J. Wang J. L. (2019). Inhibition of lncRNA HULC improves hepatic fibrosis and hepatocyte apoptosis by inhibiting the MAPK signaling pathway in rats with nonalcoholic fatty liver disease. J. Cell Physiol. 234 (10), 18169–18179. 10.1002/jcp.28450 30908654

[B47] Shin J. H. Lee C. W. Oh S. J. Yun J. Kang M. R. Han S. B. (2014). Hepatoprotective effect of aged black garlic extract in rodents. Toxicol. Res. 30 (1), 49–54. 10.5487/TR.2014.30.1.049 24795800 PMC4007044

[B48] Shu Y. Y. Gao W. K. Chu H. K. Yang L. Pan X. L. Ye J. (2022). Attenuation by time-restricted feeding of high-fat and high-fructose diet-induced NASH in mice is related to Per2 and ferroptosis. Oxid. Med. Cell Longev. 2022, 8063897. 10.1155/2022/8063897 36285301 PMC9588383

[B49] Soleimani D. Moosavian S. P. Zolfaghari H. Paknahad Z. (2021). Effect of garlic powder supplementation on blood pressure and hs-C-reactive protein among nonalcoholic fatty liver disease patients: a randomized, double-blind, placebo-controlled trial. Food Sci. Nutr. 9 (7), 3556–3562. 10.1002/fsn3.2307 34262716 PMC8269577

[B50] Stockwell B. R. Angeli J. P. F. Bayir H. Bush A. I. Conrad M. Dixon S. J. (2017). Ferroptosis: a regulated cell death nexus linking metabolism, redox biology, and disease. Cell 171 (2), 273–285. 10.1016/j.cell.2017.09.021 28985560 PMC5685180

[B51] Takahashi Y. Fukusato T. (2014). Histopathology of nonalcoholic fatty liver disease/nonalcoholic steatohepatitis. World J. Gastroentero 20 (42), 15539–15548. 10.3748/wjg.v20.i42.15539 25400438 PMC4229519

[B52] Tang D. L. Chen X. Kang R. Kroemer G. (2021). Ferroptosis: molecular mechanisms and health implications. Cell Res. 31 (2), 107–125. 10.1038/s41422-020-00441-1 33268902 PMC8026611

[B53] Targher G. Bertolini L. Padovani R. Rodella S. Tessari R. Zenari L. (2007). Prevalence of nonalcoholic fatty liver disease and its association with cardiovascular disease among type 2 diabetic patients. Diabetes Care 30 (5), 1212–1218. 10.2337/dc06-2247 17277038

[B54] Tsurusaki S. Tsuchiya Y. Koumura T. Nakasone M. Sakamoto T. Matsuoka M. (2019). Hepatic ferroptosis plays an important role as the trigger for initiating inflammation in nonalcoholic steatohepatitis. Cell Death Dis. 10 (6), 449. 10.1038/s41419-019-1678-y 31209199 PMC6579767

[B55] Utama G. L. Rahmi Z. Sari M. P. Hanidah I. I. (2024). Psychochemical changes and functional properties of organosulfur and polysaccharide compounds of Black garlic (Allium sativum L.). Curr. Res. Food Sci. 8, 100717. 10.1016/j.crfs.2024.100717 38559380 PMC10978486

[B56] Vilar-Gomez E. Martinez-Perez Y. Calzadilla-Bertot L. Torres-Gonzalez A. Gra-Oramas B. Gonzalez-Fabian L. (2015). Weight loss through lifestyle modification significantly reduces features of nonalcoholic steatohepatitis. Gastroenterology 149 (2), 367–e15. 10.1053/j.gastro.2015.04.005 25865049

[B57] Wang X. H. Zhang X. Zhang D. J. (2023). Enhancement of cation exchange and glucose binding capacity, flavonoids release and antioxidant capacity of tartary buckwheat powder with ultrafine grinding. Front. Nutr. 10, 1276017. 10.3389/fnut.2023.1276017 37927498 PMC10620305

[B58] Wei S. Qiu T. M. Wang N. N. Yao X. F. Jiang L. P. Jia X. (2020). Ferroptosis mediated by the interaction between Mfn2 and IREα promotes arsenic-induced nonalcoholic steatohepatitis. Environ. Res. 188, 109824. 10.1016/j.envres.2020.109824 32593899

[B59] Wei W. C. Wong C. C. Jia Z. J. Liu W. X. Liu C. A. Ji F. F. (2023). Parabacteroides distasonis uses dietary inulin to suppress NASH *via* its metabolite pentadecanoic acid. Nat. Microbiol. 8 (8), 1534–1548. 10.1038/s41564-023-01418-7 37386075 PMC10390331

[B60] Winn N. C. Liu Y. Rector R. S. Parks E. J. Ibdah J. A. Kanaley J. A. (2018). Energy-matched moderate and high intensity exercise training improves nonalcoholic fatty liver disease risk independent of changes in body mass or abdominal adiposity - a randomized trial. Metabolism 78, 128–140. 10.1016/j.metabol.2017.08.012 28941598

[B61] Wong V. W. S. Chan R. S. M. Wong G. L. H. Cheung B. H. K. Chu W. C. W. Yeung D. K. W. (2013). Community-based lifestyle modification programme for non-alcoholic fatty liver disease: a randomized controlled trial. J. Hepatol. 59 (3), 536–542. 10.1016/j.jhep.2013.04.013 23623998

[B62] Wu Z. R. Chen P. Li Y. Li J. Y. Wang X. Wang Y. (2015). Two cinnamoyloctopamine antioxidants from garlic skin attenuates oxidative stress and liver pathology in rats with non-alcoholic steatohepatitis. Phytomedicine 22 (1), 178–182. 10.1016/j.phymed.2014.11.013 25636888

[B63] Xiao J. Ching Y. P. Liong E. C. Nanji A. A. Fung M. L. Tipoe G. L. (2013a). Garlic-derived S-allylmercaptocysteine is a hepato-protective agent in non-alcoholic fatty liver disease *in vivo* animal model. Eur. J. Nutr. 52 (1), 179–191. 10.1007/s00394-012-0301-0 22278044 PMC3549410

[B64] Xiao J. Guo R. Fung M. L. Liong E. C. Chang R. C. C. Ching Y. P. (2013b). Garlic-Derived S-Allylmercaptocysteine ameliorates nonalcoholic fatty liver disease in a rat model through inhibition of apoptosis and enhancing autophagy. Evid. Based Complement. Altern. Med. 2013, 642920. 10.1155/2013/642920 23861709 PMC3703729

[B65] Xie Y. Hou W. Song X. Yu Y. Huang J. Sun X. (2016). Ferroptosis: process and function. Cell Death Differ. 23 (3), 369–379. 10.1038/cdd.2015.158 26794443 PMC5072448

[B66] Yan C. Yang Q. Q. Shen H. M. Spitsbergen J. M. Gong Z. Y. (2017). Chronically high level of tgfb1a induction causes both hepatocellular carcinoma and cholangiocarcinoma *via* a dominant erk pathway in zebrafish. ONCOTARGET 8 (44), 77096–77109. 10.18632/oncotarget.20357 29100373 PMC5652767

[B67] Yan T. T. Yan N. N. Wang P. Xia Y. L. Hao H. P. Wang G. J. (2020). Herbal drug discovery for the treatment of nonalcoholic fatty liver disease. Acta Pharm. Sin. B 10 (1), 3–18. 10.1016/j.apsb.2019.11.017 31993304 PMC6977016

[B68] Yang W. S. Stockwell B. R. (2016). Ferroptosis: death by lipid peroxidation. Trends Cell Biol. 26 (3), 165–176. 10.1016/j.tcb.2015.10.014 26653790 PMC4764384

[B69] Younossi Z. Anstee Q. M. Marietti M. Hardy T. Henry L. Eslam M. (2018). Global burden of NAFLD and NASH: trends, predictions, risk factors and prevention. Nat. Rev. Gastro Hepat. 15 (1), 11–20. 10.1038/nrgastro.2017.109 28930295

[B70] Yun Y. R. Lee J. E. (2022). Alliin, capsaicin, and gingerol attenuate endoplasmic reticulum stress-induced hepatic steatosis in HepG2 cells and C57BL/6N mice. J. Funct. Foods 95, 105186. 10.1016/j.jff.2022.105186 38367426

[B71] Zhang C. He X. Sheng Y. Xu J. Yang C. Zheng S. (2020). Allicin regulates energy homeostasis through brown adipose tissue. iScience 23 (5), 101113. 10.1016/j.isci.2020.101113 32413611 PMC7226876

[B72] Zhang F. Jin H. H. Wu L. Shao J. J. Zhu X. J. Chen A. P. (2017). Diallyl trisulfide suppresses oxidative stress-induced activation of hepatic stellate cells through production of hydrogen sulfide. Oxid. Med. Cell Longev. 2017, 1406726. 10.1155/2017/1406726 28303169 PMC5337887

[B73] Zhao X. Y. Yang Z. B. Gai G. S. Yang M. (2009). Effect of superfine grinding on properties of ginger powder. J. Food Eng. 91 (2), 217–222. 10.1016/j.jfoodeng.2008.08.024

