# Supplementary material for: Ultrafine garlic powder alleviates non-alcoholic steatohepatitis by inhibiting hepatocyte ferroptosis and modulating ERK-dependent oxidative stress
Source: Front Pharmacol. 2025 Nov 19;16:1711917. doi: 10.3389/fphar.2025.1711917 (PMC12673841; doi:10.3389/fphar.2025.1711917)
Supplement: Supplementary file 1 [file Supplementaryfile1.docx]

**Supplementary figures**

**
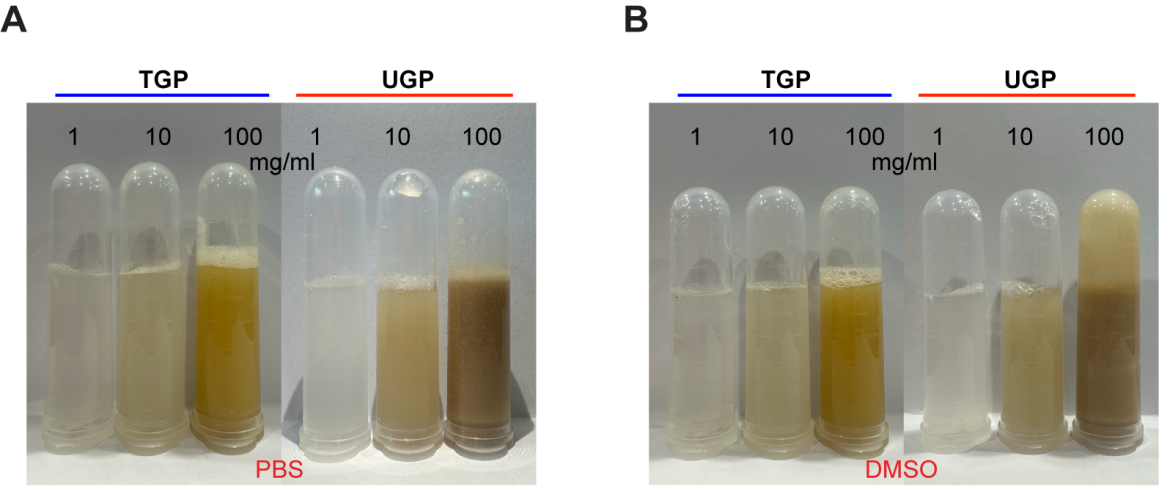
**

**Supplementary Figure S1. Solubility and dispersibility of TGP and UGP.** (A) Solubility and dispersibility of TGP and UGP in PBS. (B) Solubility and dispersibility of TGP and UGP in DMSO.（TGP: Traditional garlic powder; UGP: Ultrafine garlic powder.)

**
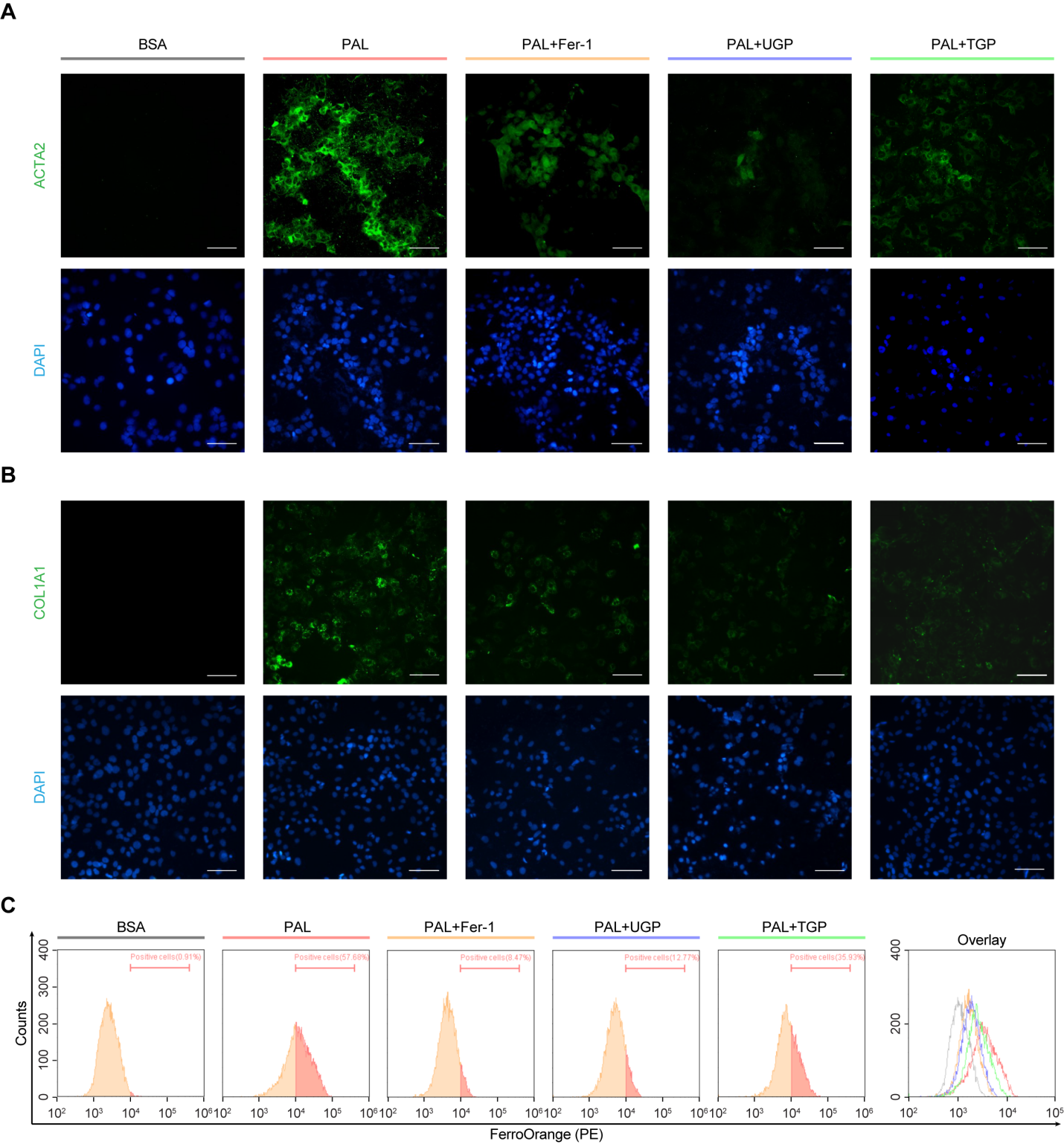
**

**Supplementary Figure S2. Effects of UGP on cellular Fe^2+^ levels in lipotoxic hepatocytes and activation of HSCs.** (A) Representative immunofluorescent images of ACTA2 (green) and DAPI (blue) in LX-2 cells (scale bar = 100 μm). (B) Representative immunofluorescent images of COL1A1 (green) and DAPI (blue) in LX-2 cells (scale bar = 100 μm). (C) Flow cytometry analysis of cellular Fe^2+^ levels by FerroOrange staining on THLE2 cells. (ACTA2: Actin alpha cardiac muscle 2; COL1A1: Collagen type I alpha 1 chain; PAL: Palmitic acid; TGP: Traditional garlic powder; UGP: Ultrafine garlic powder.)

**
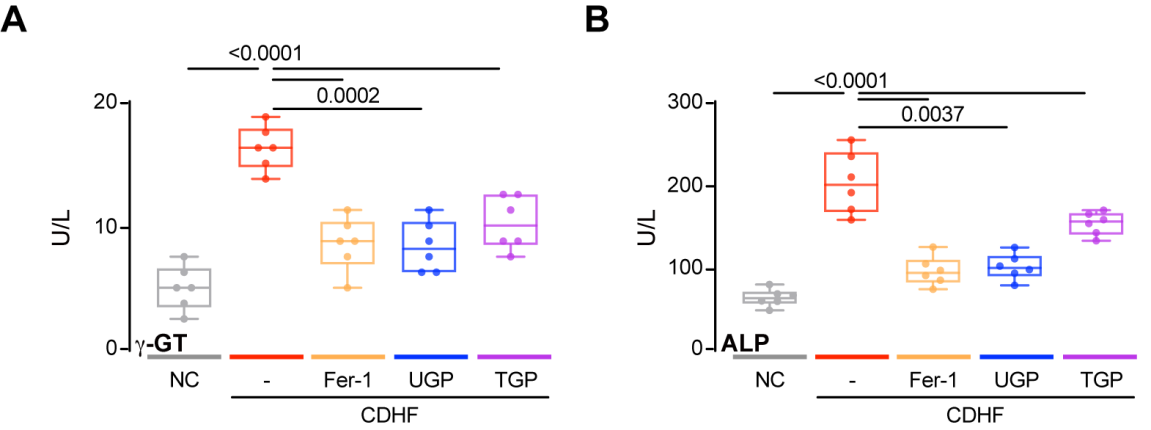
**

**Supplementary Figure S3. UGP protects against liver damage in CDHF-induced acute liver injury model.** (A) Serum γ-GT levels (n = 6). (B) Serum ALP levels (n = 6). Data are shown as box-and-whisker with median (middle line), 25th-75th percentiles (box) and min-max values (whiskers); one-way ANOVA with Tukey’s correction. (ALT: Alanine transaminase; γ-GT, γ-Glutamyltransferase; TGP: Traditional garlic powder ;UGP: Ultrafine garlic powder.)

**
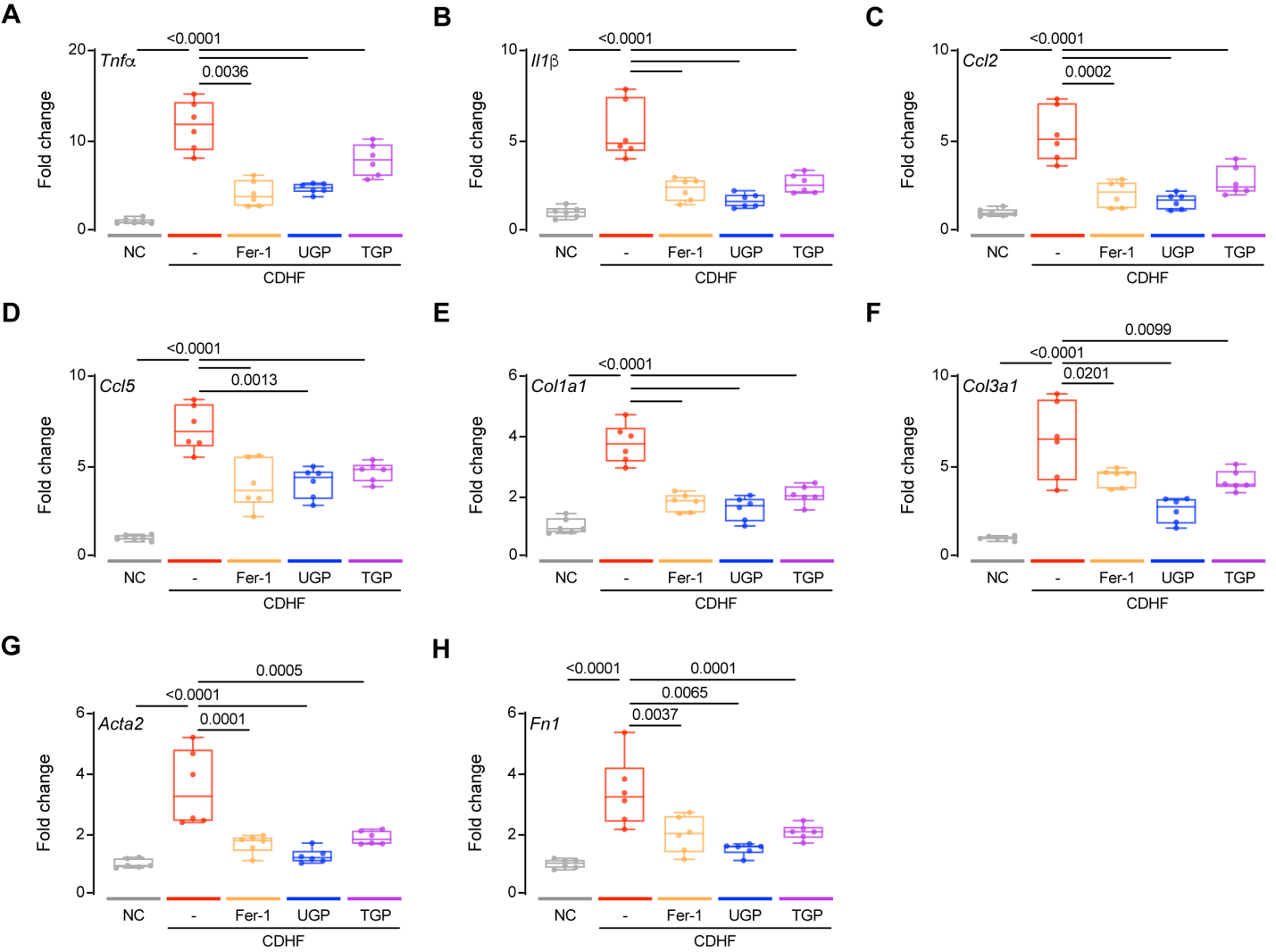
**

**Supplementary Figure S4. UGP protects against liver inflammation and fibrosis in CDHF-induced acute liver injury model.** Hepatic mRNA expression of pro-inflammatory markers *Tnfα* (A, n = 6), *Il1β* (B, n = 6), *Ccl2* (C, n = 6), and *Ccl5* (D, n = 6), and fibrotic markers *Col1a1* (E, n = 6), *Col3a1* (F, n = 6), *Acta2* (G, n = 6), and *Fn1* (H, n = 6) as shown in Fig. 4K. Data are shown as box-and-whisker with median (middle line), 25th-75th percentiles (box) and min-max values (whiskers); one-way ANOVA with Tukey’s correction. (ACTA2: Actin alpha cardiac muscle 2; CCL2: C-C motif chemokine ligand 2; CCL5: C-C motif chemokine ligand 5; COL1A1: Collagen type I alpha 1 chain; COL3A1: Collagen type III alpha 1 chain; FN: Fibronectin; IL-1β: Interleukin 1 beta; NASH: Non-alcoholic steatohepatitis; TGP: Traditional garlic powder ;TNF-α: Tumor necrosis factor alpha. UGP: Ultrafine garlic powder.)

**
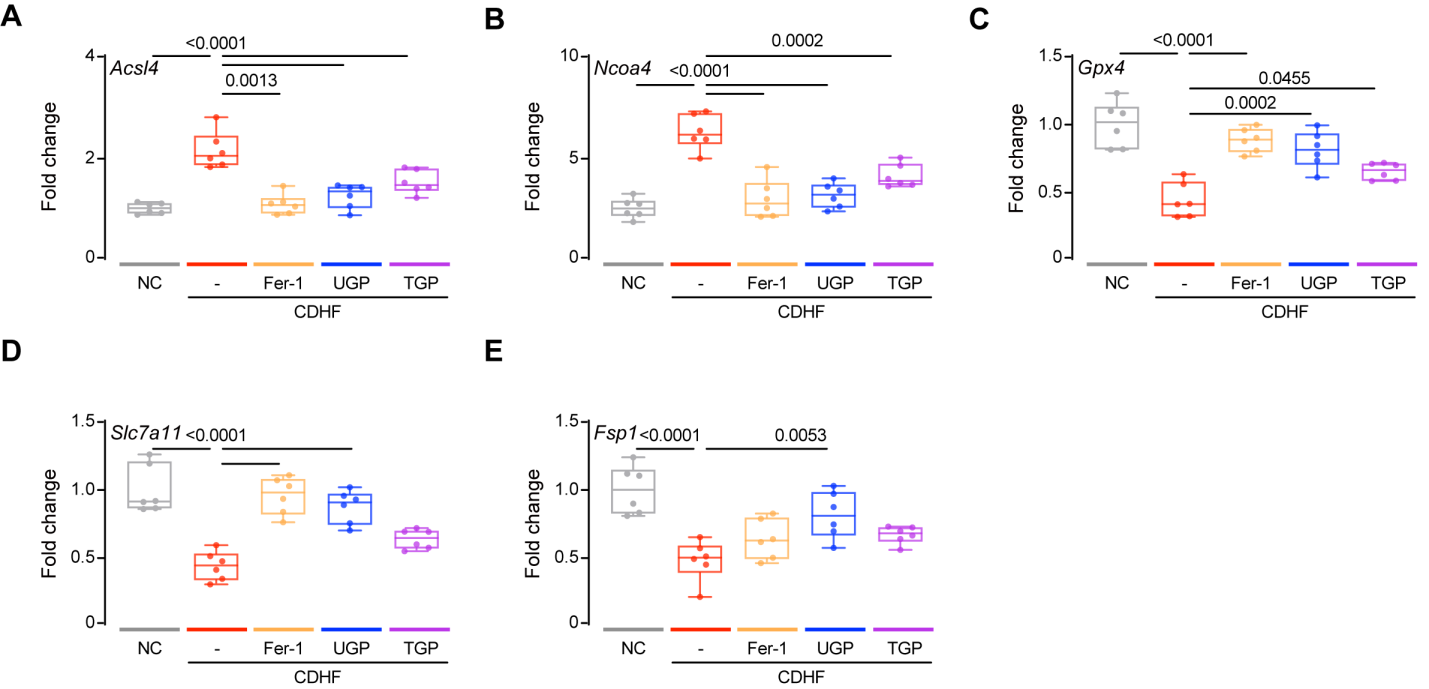
**

**Supplementary Figure S5. UGP protects against liver ferroptosis in CDHF-induced acute liver injury model.** Hepatic mRNA expression of ferroptosis markers *Acsl4* (A, n = 6), *Ncoa4* (B, n = 6), *Gpx4* (C, n = 6), *Slc7a11* (D, n = 6), and *Fsp1* (E, n = 6) as shown in Fig. 5D. Data are shown as box-and-whisker with median (middle line), 25th-75th percentiles (box) and min-max values (whiskers); one-way ANOVA with Tukey’s correction. (Acsl4: Acyl-CoA Synthetase Long Chain Family Member 4；FSP1: Ferroptosis Suppressor Protein 1；GPX4: Glutathione Peroxidase 4；NCOA4: Nuclear Receptor Coactivator 4；SLC7A11: Solute Carrier Family 7 Member 11；TGP: Traditional garlic powder ;UGP: Ultrafine garlic powder.)

**
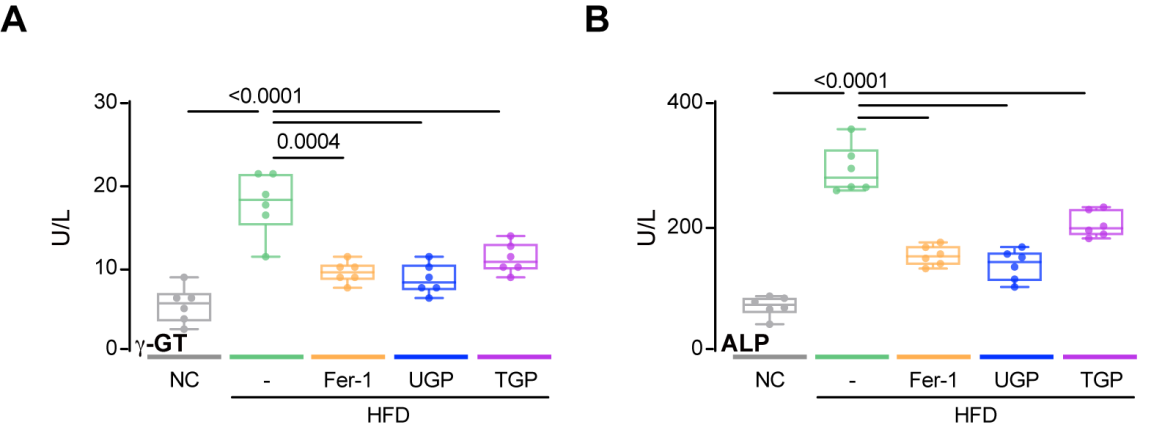
**

**Supplementary Figure S6. UGP protects against liver damage in HFD-induced chronic liver injury model.** (A) Serum γ-GT levels (n = 6). (B) Serum ALP levels (n = 6). Data are shown as box-and-whisker with median (middle line), 25th-75th percentiles (box) and min-max values (whiskers); one-way ANOVA with Tukey’s correction. (ALT: Alanine transaminase; γ-GT, γ-Glutamyltransferase; TGP: Traditional garlic powder ;UGP: Ultrafine garlic powder.)

**
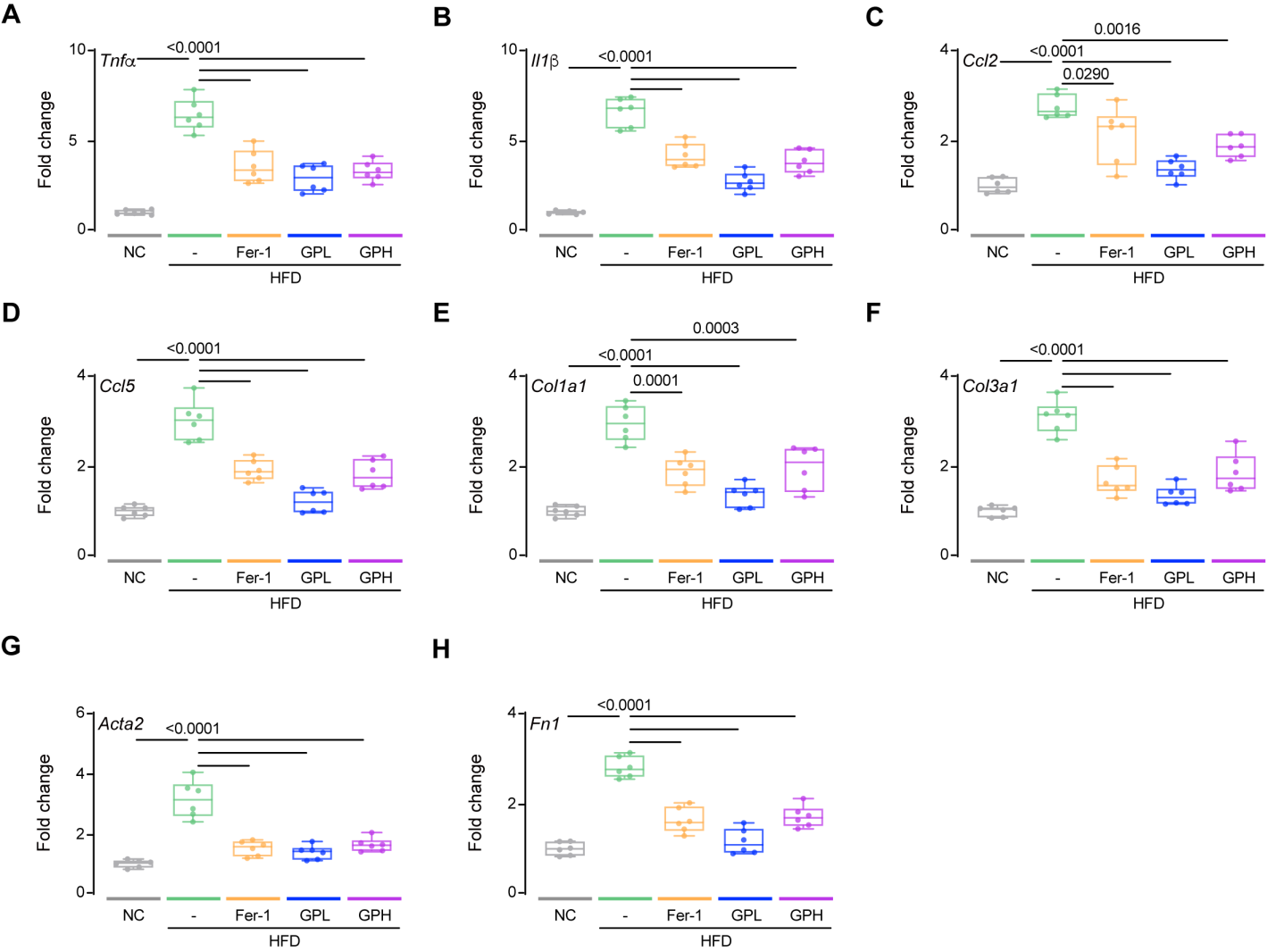
**

**Supplementary Figure S7. UGP protects against liver inflammation and fibrosis in HFD-induced chronic liver injury model.** Hepatic mRNA expression of pro-inflammatory markers *Tnfα* (A, n = 6), *Il1β* (B, n = 6), *Ccl2* (C, n = 6), and *Ccl5* (D, n = 6), and fibrotic markers *Col1a1* (E, n = 6), *Col3a1*(F, n = 6), *Acta2* (G, n = 6), and *Fn1* (H, n = 6) as shown in Fig. 6K. Data are shown as box-and-whisker with median (middle line), 25th-75th percentiles (box) and min-max values (whiskers); one-way ANOVA with Tukey’s correction. (ACTA2: Actin alpha cardiac muscle 2; CCL2: C-C motif chemokine ligand 2; CCL5: C-C motif chemokine ligand 5; COL1A1: Collagen type I alpha 1 chain; COL3A1: Collagen type III alpha 1 chain; FN: Fibronectin; IL-1β: Interleukin 1 beta; NASH: Non-alcoholic steatohepatitis; TGP: Traditional garlic powder ;TNF-α: Tumor necrosis factor alpha. UGP: Ultrafine garlic powder.)


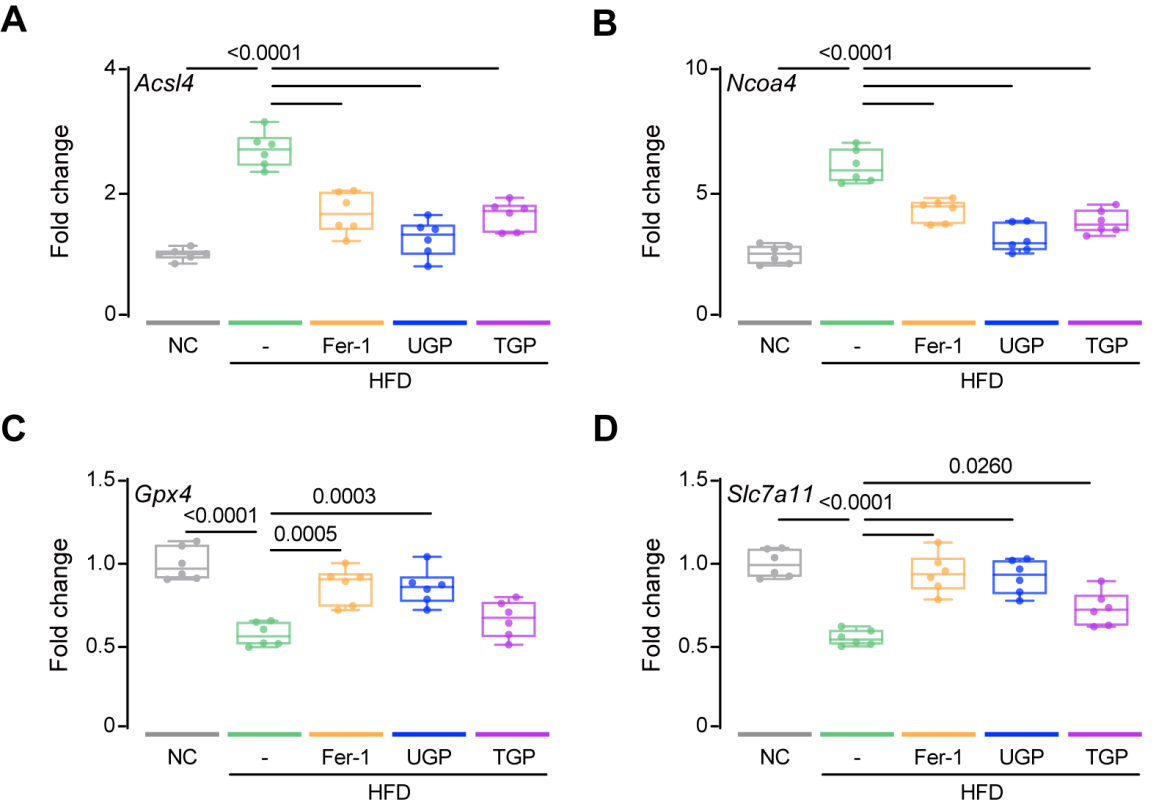


**Supplementary Figure S8. UGP protects against liver ferroptosis in HFD-induced acute liver injury model.** Hepatic mRNA expression of ferroptosis markers *Acsl4* (A, n = 6), *Ncoa4* (B, n = 6), *Gpx4* (C, n = 6), and *Slc7a11* (D, n = 6) as shown in Fig. 8D. Data are shown as box-and-whisker with median (middle line), 25th-75th percentiles (box) and min-max values (whiskers); one-way ANOVA with Tukey’s correction. (Acsl4: Acyl-CoA Synthetase Long Chain Family Member 4；FSP1: Ferroptosis Suppressor Protein 1；GPX4: Glutathione Peroxidase 4；NCOA4: Nuclear Receptor Coactivator 4；SLC7A11: Solute Carrier Family 7 Member 11；TGP: Traditional garlic powder ;UGP: Ultrafine garlic powder.)
